# Antibacterial and Cytotoxic New Napyradiomycins from the Marine-Derived *Streptomyces* sp. SCSIO 10428

**DOI:** 10.3390/md11062113

**Published:** 2013-06-14

**Authors:** Zhengchao Wu, Sumei Li, Jie Li, Yuchan Chen, Kumar Saurav, Qingbo Zhang, Haibo Zhang, Wenjun Zhang, Weimin Zhang, Si Zhang, Changsheng Zhang

**Affiliations:** 1 Key Laboratory of Marine Bio-resources Sustainable Utilization, RNAM Center for Marine Microbiology, Guangdong Key Laboratory of Marine Materia Medica, South China Sea Institute of Oceanology, Chinese Academy of Sciences, 164 West Xingang Road, Guangzhou 510301, China; E-Mails: charleszcwu@gmail.com (Z.W.); lism@scsio.ac.cn (S.L.); lijietaren@scsio.ac.cn (J.L.); sauravverma17@gmail.com (K.S.); gudaobo@163.com (Q.Z.); zhanghb@scsio.ac.cn (H.Z.); wenjunzha@gmail.com (W.Z.); zhsimd@scsio.ac.cn (S.Z.); 2 University of Chinese Academy of Sciences, Beijing 100049, China; 3 Guangdong Institute of Microbiology, 100 Central Xianlie Road, Guangzhou 510070, China; E-Mails: yuchan2006@126.com (Y.C.); wmzhang58@yahoo.com.cn (W.Z.)

**Keywords:** napyradiomycins, marine actinomycetes, natural products, antibacterial, cytotoxicity

## Abstract

Three new napyradiomycins (**1**–**3**) were isolated from the culture broth of a marine-derived actinomycete strain SCSIO 10428, together with six known related analogues napyradiomycin A1 (**4**), 18-oxonapyradiomycin A1 (**5**), napyradiomycin B1 (**6**), napyradiomycin B3 (**7**), naphthomevalin (**8**), and napyradiomycin SR (**9**). The strain SCSIO 10428 was identified as a *Streptomyces* species by the sequence analysis of its 16S rRNA gene. The structures of new compounds **1**–**3**, designated 4-dehydro-4a-dechloronapyradiomycin A1 (**1**), 3-dechloro-3-bromonapyradiomycin A1 (**2**), and 3-chloro-6,8-dihydroxy-8-α-lapachone (**3**), respectively, were elucidated by comparing their 1D and 2D NMR spectroscopic data with known congeners. None of the napyradiomycins **1**–**9** showed antioxidative activities. Napyradiomycins **1**–**8** displayed antibacterial activities against three Gram-positive bacteria *Staphylococcus* and *Bacillus* strains with MIC values ranging from 0.25 to 32 μg mL^−1^, with the exception that compound **3** had a MIC value of above 128 μg mL^−1^ against *Staphylococcus aureus* ATCC 29213. Napyradiomycins **2**, **4**, **6**, and **7** exhibited moderate cytotoxicities against four human cancer cell lines SF-268, MCF-7, NCI-H460, and HepG-2 with IC_50_ values below 20 μM, while the IC_50_ values for other five napyradiomycins **1**, **3**, **5**, **8** and **9** were above 20 μM.

## 1. Introduction

In the past decade, marine-derived actinomycetes have emerged as a valuable source for drug discovery [1,2]. A number of structurally unique natural products with antitumor [3,4], antiinfective [5], and antimalarial bioactivities [6], have been discovered from marine-derived actinomycetes. Our recent efforts on marine-derived actinomycetes also led to the isolation of structurally diverse novel natural products with antibacterial, antitumor, and antimalarial bioactivities [7,8,9,10,11,12,13,14,15,16,17].

In the course of our continuous searching for new bioactive natural products from marine-derived actinomycetes, crude fermentation extracts of the actinomycetal strain SCSIO 10428 were found to exhibit significant antimicrobial activities and to be lethal to brine shrimp (*Artemia salina*). Large scale fermentation of the strain SCSIO 10428 led to the isolation of nine napyradiomycin type natural products. Herein we report the isolation, structure elucidation, and biological activities of these nine compounds.

## 2. Results and Discussion

### 2.1. Strain Identification and Compounds Isolation

**Figure 1:**
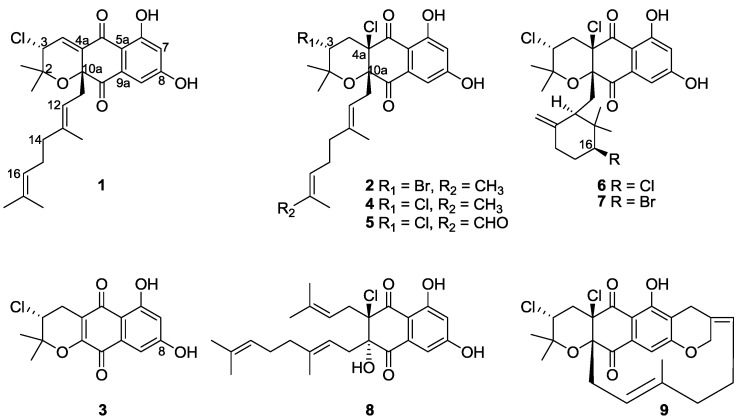
Chemical structures of compounds **1**–**9**.

The strain SCSIO 10428 was isolated from a sediment sample collected from the Xieyang island, Beihai, Guangxi province, China. The strain was identified as a member of *Streptomyces* on the basis of phylogenetic analysis. The neighbor-joining tree was constructed based on the 16S rRNA gene sequences of strain SCSIO 10428 and other closely related *Streptomyces* species (Figure S1). Purification of the EtOAc extracts of a 20 L fermentation culture of *Streptomyces* sp. SCSIO 10428 led to the isolation of nine napyradiomycin type natural products (Figure 1). Compounds **1**–**3** were elucidated as new compounds, designated 4-dehydro-4a-dechloronapyradiomycin A1 (**1**), 3-dechloro-3-bromonapyradiomycin A1 (**2**), and 3-chloro-6,8-dihydroxy-8-α-lapachone (**3**), respectively, on the basis of the analysis of MS, 1D and 2D NMR data and the comparison with known compounds. Compounds **4**–**9** were identified as napyradiomycin A1 (**4**), 18-oxonapyradiomycin A1 (**5**), napyradiomycin B1 (**6**), napyradiomycin B3 (**7**), naphthomevalin (**8**), and napyradiomycin SR (**9**), respectively (Table S1, Supporting Information), by comparing their MS, ^1^H and ^13^C NMR spectroscopic data with those previously reported [18,19,20].

### 2.2. Structure Elucidation

**Figure 2:**
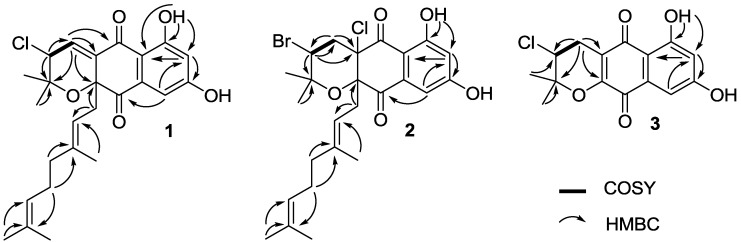
Key HMBCand COSY correlations of **1**–**3**.

Compound **1** was isolated as a pale yellow oil. The molecular formula of **1** was determined as C_25_H_29_ClO_5_ by HRESIMS (*m/z* 467.1595 [M + Na]^+^, calcd 467.1596), indicating nine degrees of unsaturation. The IR spectrum of **1** showed broad absorption bands at around 3294 cm^−1^ for multiple hydroxyl groups, and at 1701 cm^−1^ for conjugated carbonyl functionality. The strong UV absorption pattern at 257, 312 and 362 nm indicated the aromatic nature of compound **1** (Figure S2). The ^1^H NMR spectrum of **1** clearly displayed one exchangeable OH signal (δ_H_ 12.57), three single aromatic protons (δ_H_ 7.19, 6.92 and 6.71), two olefinic proton signals at δ_H_ 5.02 and 4.98, one methine proton adjacent to chlorine (δ_H_ 4.39), three methylene proton signals (δ_H_ 2.48, 2.00 and 1.94), and five quaternary aliphatic methyl groups at δ_H_ 1.68, 1.58, 1.53, 1.44 and 1.09 (Figure S2). The ^13^C NMR spectrum of **1** exhibited two carbonyl signals at δ_C_ 195.4 and 188.6, two phenolic hydroxyl groups (δ_C_ 165.5 and 164.0), ten double-bond methines or quaternary carbon signals resonating between δ_C_ 141.7 and 108.4, one quaternary carbon adjacent to oxygen atom (δ_C_ 83.3), and one methine carbon adjacent to chlorine (δ_C_ 59.6), as well as other eight aliphatic methylene or methyl carbon signals with chemical shifts below δ_C_ 40.0 (Table 1, Figure S2). These NMR spectroscopic data suggested that compound **1** was structurally related to the napyradiomycins family of antibiotics [18]. The comparison of NMR spectroscopic data of **1** and napyradiomycin A1 (**4**) (Table S1) revealed that **1** only differed from **4** by having an olefinic bond at C-4 and C-4a, which was evident from the presence of an alkene proton signal at δ_H_ 6.92 (H-4 in **1**), and the absence of the methylene proton signals at δ_H_ 2.48 and 2.41 (H_2_-4 in **4**). Similarly, the ^13^C NMR spectrum of **1** showed two olefinic carbons for C-4 (δ_C_ 136.9, CH) and C-4a (δ_C_ 137.1, C). While in the ^13^C NMR spectrum of **4** (Table S1), there was a methylene carbon at C-4 (δ_C_ 42.9, CH_2_) and a quaternary carbon at C-4a (δ_C_ 79.2, C). In support of the structure assignment of **1**, HMBC correlations were observed from H-4 to C-4a/C-5/C-10a/C-2, and from H-3 to C-4 (Figure 2, Figure S2). Therefore, the planar structure of **1** was elucidated as 4-dedydro-4a-dechloronapyradiomycin A1 (Figure 1). Although our current experimental data could not assign the stereochemistry for compound **1**, we assumed the absolute configuration of **1** as (3*R*,10a*R*) on the basis of two considerations: (i) all napyradiomycins, described to date, have been found to take a (3*R*,10a*R*) configuration [18,19,20,21,22,23,24]; (ii) biosynthetically, compound **1** was putatively derived from **4** by dechlorination, thus retaining the same configuration at C-3 [25].

**Table 1:** ^1^H, ^13^C NMRspectroscopic data of napyradiomycins **1**–**3** (Measured in CDCl_3_, at 500 MHz for ^1^H and 125 MHz for ^13^C with reference to the solvent signals, δ in ppm).

No.	1		2		3	
	δ_C_	δ_H_ multi (*J* in Hz)	δ_C_	δ_H_ multi (*J* in Hz)	δ_C_	δ_H_ multi (*J* in Hz)
2	76.9		78.7		80.7	
2-CH_3_	20.4	1.09 s	23.6	1.26 s	22.4	1.51 s
2-CH_3_	27.4	1.53 s	29.5	1.50 s	25.8	1.53 s
3	59.6	4.39 d (2.0)	51.0	4.55 dd (8.0, 8.0)	57.9	4.08 dd (5.5, 7.0)
4	136.9	6.92 d (2.0)	43.9	2.58 br d (8.0)	27.2	2.87 dd (7.0, 19.0)
						3.10 dd (5.5, 19.0)
4a	137.1		79.7		117.8	
5	188.6		193.9		188.0	
5a	112.0		110.5		108.8	
6	165.5		165.0		162.5	
6-OH		12.57 s		11.84 s		12.30 s
7	109.2	6.71 s	109.7	6.73 d (2.0)	108.9	6.62, d (2.0)
8	164.0		163.7		163.9	
9	108.4	7.19 s	107.9	7.22 d (2.0)	109.3	7.14 d (2.0)
9a	136.0		135.6		133.0	
10	195.4		196.2		178.8	
10a	83.3		83.9		153.7	
11	39.9	2.48 d (7.5)	41.6	2.70 d (8.0)		
				2.69 d (8.0)		
12	115.9	4.98 br t (7.5)	115.0	4.70 br t (8.0)		
13	141.7		143.0			
13-CH_3_	16.7	1.44 s	16.6	1.31 s		
14	39.8	1.94 m	39.9	1.60 m		
15	26.6	2.00 m	26.1	1.60 m		
16	124.0	5.02 m	123.9	4.89 br s		
17	132.1		131.9			
17-CH_3_	18.0	1.58 s	17.7	1.52 s		
17-CH_3_	25.9	1.68 s	25.8	1.62 s		

Compound **2** was obtained as pale yellow oil. The molecular formula of **2** was determined as C_25_H_31_ClBrO_5_ by HRESIMS (*m/z* 525.1047 [M + H]^+^, calcd 525.1038), indicating eight degrees of unsaturation (Figure S3). NMR comparison indicated that compounds **2** and **4** (Table 1, Table S1) only differed in the substitution at C-3. The different substitution was evident from the variations in both the carbon and proton shifts at C-3 (**4**: δ_C_ 59.0, δ_H_ 4.43, dd, *J* = 4.5 and 11.5 Hz; **2**: δ_C_ 51.0, δ_H_ 4.55, dd, *J* = 8.0 and 8.0 Hz). Therefore, compound **2** was deduced as 3-dechloro-3-bromonapyradiomycin A1 (Figure 1). The structure of **2** was further confirmed by 2D NMR spectroscopic data, such as the key HMBC correlations from H-3 to C2/C-4/C4a (Figure 2, Figure S3). Examples of bromo-substitution at C-3 for napyradiomycins have been described in a doctoral thesis [24], without any reports in the primary literatures. The absolute configuration of **2** (3*R*,4a*R*,10a*R*) was determined to be the same as that of **4** as both of their sodium D line rotation [α]_D_ values were positive (for **2**, 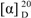 +23°, *c* 0.30 in MeOH); for **4**, 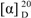 +51°, *c* 0.3 in MeOH).

Compound **3** was obtained as orange needles. Its molecular formula was determined as C_15_H_14_ClO_5_ by HRESIMS (*m/z* 309.0532 [M + H]^+^, calcd 309.0524), indicating seven degrees of unsaturation. Compound **3** showed UV absorption bands at 263, 305 and 450 nm, indicating a naphthoquinone chromophore (Figure S4). NMR comparison revealed that **3** was only different from (*R*)-3-chloro-6-hydroxy-8-methoxy-α-lapachone by the lack of signals for a methoxy group (δ_C_ 56.0, δ_H_ 3.87) at C-8 [19], indicating that **3** was 3-chloro-6,8-dihydroxy-α-lapachone. The structure of **3** was further confirmed by 2D NMR correlations (Figure 2, Figure S4, Figure S5). The absolute configuration of **3** was assigned as 3*R*, on the basis of the similar sodium D line rotation [α]_D_ values of **3** (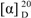 −22°, *c* 0.14 in CHCl_3_) and (*R*)-3-chloro-6-hydroxy-8-methoxy-α-lapachone (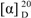 −8°, *c* 0.12 in CHCl_3_).

### 2.3. Biological Activities

Napyradiomycins **1**–**9** were evaluated for their antibacterial, cytotoxic and antioxidative activities [26]. Napyradiomycins **1**–**8** showed antibacterial activities with MIC values ranging from 0.5 to 32 μg mL^−1^ against three Gram-positive bacteria *Staphylococcus aureus* ATCC 29213, *Bacillus subtilis* SCSIO BS01 and *Bacillus thuringensis* SCSIO BT01, except that compound **3** had no antibacterial activity (MIC > 128 μg mL^−1^) against *S. aureus* ATCC 29213 (Table 2). No compounds exhibited activity against the Gram-negative bacterium *Escherichia coli* ATCC 25922 (Table 2). The main product napyradiomycin A1 (**4**) displayed comparable antibacterial activities with the positive control ampicillin with MIC values of 1–2 μg mL^−1^, while the new compound **2** (MIC values of 0.5–1 μg mL^−1^) was slightly better than **4** in antibacterial potency. Napyradiomycin B3 (**7**) exhibited the best antibacterial activities (MIC values of 0.25–0.5 μg mL^−1^) among these nine napyradiomycins.

In cytotoxicity assays against four human cancer cell lines SF-268, MCF-7, NCI-H460, and HepG-2, four napyradiomycins **2**, **4**, **6** and **7** showed moderate cytotoxic activities with IC_50_ values below 20 μM (Table 2), and the other five napyradiomycins **1**, **3**, **5**, **8** and **9** had neglectible cytotoxicities with IC_50_ values above 20 μM against these four cancer cell lines.

The antioxidant activity of napyradiomycins **1**–**9** was evaluated by DPPH (2,2-diphenyl-1-picryl-hydrazyl-hydrate) radical scavenging activity assays and compared with that of the reference antioxidant Trolox [27]. However, none of the napyradiomycins **1**–**9** exhibited detectable antioxidant activities.

**Table 2:** Antibacterial and cytotoxic activities of compounds **1**–**9**.

	MIC (μg mL^−1^)				IC_50_ (μM)			
	*Sa*	*Bs*	*Bt*	*Ec*	SF-268	MCF-7	NCI-H460	HepG-2
**1**	4	4	8	>128	22.8 ± 0.3	20.6 ± 0.1	22.4 ± 0.1	21.8 ± 0.5
**2**	0.5	1	1	>128	11.5 ± 1.2	16.2 ± 0.7	18.1 ± 0.3	17.1 ± 1.0
**3**	>128	8	16	>128	23.8 ± 2.2	71.1 ± 0.4	127.1 ± 0.9	59.4 ± 0.7
**4**	1	2	2	>128	18.5 ± 0.3	9.8 ± 0.2	19.0 ± 1.0	18.9 ± 0.3
**5**	32	8	16	>128	132.7 ± 0.1	138.2 ± 0.8	137.1 ± 1.5	149.1 ± 1.4
**6**	1	2	0.5	>128	11.1 ± 0.1	17.0 ± 0.2	18.6 ± 0.4	17.9 ± 0.7
**7**	0.5	0.25	0.5	>128	15.3 ± 1.1	11.2 ± 0.5	17.2 ± 0.4	10.5 ± 1.6
**8**	1	2	2	>128	29.6 ± 0.2	22.2 ± 0.7	26.9 ± 0.3	61.2 ± 0.1
**9**	>128	>128	>128	>128	98.1 ± 1.7	40.5 ± 1.4	163.7 ± 1.0	157.2 ± 4.5
CK *	2	1	2	2	7.3 ± 0.9	4.1 ± 0.3	4.4 ± 0.1	5.6 ± 0.3

Note: **CK** * stands for ampicillin in the antibacterial assays with cisplatin in the cytotoxicity assays as a positive control. *Sa*, *Staphylococcus aureus* ATCC 29213; *Bs*, *Bacillus subtilis* SCSIO BS01; *Bt*, *Bacillus thuringensis* SCSIO BT01; *Ec*, *Escherichia coli* ATCC 25922.

### 2.4. Discussion

Napyradiomycins are a large class of metabolites mainly produced by bacteria in the family Steptomycetaceae. Napyradiomycins were first isolated from terrestrial microorganisms from 1986 [18,28], and then were discovered from the marine-derived strains from the last decade [22,23]. Up to date, about 43 derivatives of napyradiomycins have been discovered [18,19,20,21,22,23,24,25,29,30,31,32,33]. Structurally, napyradiomycins consist of a semi-napthoquinone core, a prenyl unit attached at C-4a that is cyclized to form a tetrahydropyran ring in most cases, and a monoterpenoid substituent attached at C-10a. Structure variations of napyradiomycins arise mainly from the 10-carbon monoterpenoid subunit, which is linear (napyradiomycin A series), or cyclized to a 6-membered ring (napyradiomycin B series), or cyclized to a 14-membered ring (napyradiomycin C series) [18]. The different halogenation patterns also contribute largely to structure variations of napyradiomycins [24]. Biosynthetically, napyradiomycins originate from a hybrid terpenoid/polyketide pathway which is rare in nature [24,25,34,35,36].

Napyradiomycins exhibit a diverse range of biological activities, including anti-microbial activities [28,30], gastric (H^+^-K^+^) ATPases inhibiting activities [37], as estrogen receptor antagonists, as well as cytotoxicity [38], and apoptosis-inducing activities [24]. Farnaes compared several napyradiomycins B series for structure-activity relationships (SAR) in their cytotoxicities against HCT-116 colon cancer cells, and suggested that the C-3 brominated derivatives showed slightly better cytotoxicities than their C-3 chlorinated counterparts [24]. In this study, the new compound **2** with a bromine at C-3 displayed comparable potency in antibacterial and cytotoxic activities to napyradiomycin A1 (**4**) with a chlorine at C-3 (Table 2). Also, the bromination at C-16 (e.g., compound **7**) had a slightly positive effect on antibacterial potency, compared with the chlorination at C-16 (e.g., compound **6**). The dechlorination at C-4a of **4** to afford a double bond in **1** caused a slight decline in both antibacterial and cytotoxic potency (Table 2). The oxidation of one of the C-17 methyl groups in **4** generated an aldehyde group in **5**, which significantly reduced the biological activity.

## 3. Experimental Section

### 3.1. General Experimental Procedures

Optical rotations were measured on an Anton Paar MCP 500 polarimeter. UV spectra were recorded on a Hitachi U-2900 spectrophotometer. IR spectra were recorded on a Shimadzu IRAffinity-1 FT-IR spectrometer. 1D- and 2D-NMR spectra were recorded at 500 MHz (for ^1^H) and 125 MHz (for ^13^C) on a Bruker AV-500 MHz spectrometer (Bruker Biospin GmbH, Rheinstetten, Germany) in CDCl_3_ or CD_3_OD using solvent peaks as references. HRESIMS data were measured using a Bruker Maxis quadrupole-time-of-flight mass spectrometer. Open column chromatography (CC) was performed with silica-gel (100–200 mesh; Jiangyou Si-gel Development, Co., Ltd., Yantai, China), and Sephadex LH-20 (GE Healthcare Biosciences AB, Sweden). Medium pressure liquid chromatography (MPLC) was performed on automatic flash chromatography (EZ Purifier III, Leisure Science Co., Ltd., China) with a precast normal-phase column (Si-gel, 40–60 μm, 6 nm; Bonna-Agela Technologies Co., Ltd., Tianjin, China), or a reversed-phase column (YMC-Pack ODS-A, 50 μm, 12 nm). Semi-preparative HPLC separation was performed on Angilent Chemstation (1260 Infinity; Angilent Technologies, USA) or Hitachi Model D-2000 Elite station (Hitachi Technologies, Japan) with a semi-preparative reversed-phase column (Phenomenex, Luna Phenyl-Hexyl, 250 × 10 mm, 5 μm, 10 nm; or Phenomenex, Gemini C18, 250 × 10 mm, 5 μm, 11 nm). Preparative HPLC separation was performed on Clarity station (Chuang Xin Tong Heng Science and Technology Co., Ltd., Beijing, China) with a reversed-phase column (YMC-Pack ODS-A, 250 × 20 mm, 5 μm, 12 nm). Thin-layer chromatography (TLC) was carried out with glass precoated Si-gel GF254 plates.

### 3.2. Collection and Phylogenetic Analysis of Strain

The strain SCSIO 10428 was isolated from a sediment sample collected from the Xieyang island, Beihai, Guangxi province, China. The strain was identified as a species of *Streptomyces* (GenBank accession number KC899266 for its 16S rRNA gene sequence) on the basis of the phylogenetic tree constructed by a neighbor-joining method (Figure S1).

### 3.3. Fermentation

A single colony of *Streptomyces* sp. SCSIO 10428 was inoculated into 50 mL of seed medium (modified A1BFe + C) [39] [starch 10 g L^−1^, yeast extract 4 g L^−1^, peptone 2 g L^−1^, KBr 0.1 g L^−1^, Fe_2_(SO4)_3_·4H_2_O 0.04 g L^−1^, CaCO_3_ 1 g L^−1^, sea salt 30 g L^−1^, pH 7.0, adjusted before sterilization] in 250 mL Erlenmeyer flasks, and was cultured on a rotary shaker at 200 rpm and 28 °C for 3 days. Around 6% inoculums were transferred into 150 mL production medium (the same as the seed medium) in 500 mL Erlenmeyer flasks, and were subsequently incubated on a rotary shaker at 200 rpm, 28 °C for 7 days. A total of 20 L cultures were prepared for fermentation.

### 3.4. Extraction and Isolation

The 20 L fermentation broth was extracted three times with EtOAc (20 L), and the mycelia cake was extracted three times with EtOH (2 L). After removal of the organic solvent, residues both from fermentation broth and from mycelia cake were combined to afford crude extracts (16 g). The crude extracts were subjected to normal-phase Si-gel open CC (100–200 mesh, 35 g) and eluted with isooctane/EtOAc (30:1; 16:1; 8:1; 4:1; 2:1; 1:1 and 0:1, v/v, each of 400 mL) and CHCl_3_/MeOH gradient (1:1 and 0:1, v/v, each of 400 mL) to yield 9 fractions (Fr.1–Fr.9). Fr.1 (0.4 g) was first subjected to Sephadex LH-20 CC eluted with CHCl_3_/MeOH (1:1) then the third subfraction Fr.13 (121 mg) was subjected to Si-gel normal-phase MPLC (40–60 μm, 6 nm, 8 g) eluting by hexane/EtOAc to give three subfractions: Fr.131 (100:0, v/v), Fr.132 (98:2, v/v), and Fr.133 (96:4, v/v).

Compound **9** (4.0 mg) was obtained from the Fr.132 (7.4 mg) by preparative TLC (PTLC) and eluted with petroleum ether/EtOAc (96:4). Fr.3 (3 g) was purified by C18 reversed-phase MPLC (35 × 3 cm), eluting with a gradient of MeCN/H_2_O (40:60, 65:35, 80:20, 82:18, 84:16, 93:17; 96:14 and 100:0, v/v, each of 600 mL) to yield 9 subfractions (Fr.3A–Fr.3I). Fr.3I (367 mg) was subjected to Si-gel normal-phase MPLC (40–60 μm, 6 nm, 12 g) eluting by hexane/EtOAc to give three subfractions: Fr.3I1 (98:2, v/v), Fr.3I2 (96:4, v/v), and Fr.3I3 (90:10, v/v). Fr.3I2 (258 mg) was subjected to Sephadex LH-20 CC eluted with CHCl_3_/MeOH (1:1) then the second subfraction Fr.3I22 (107 mg) was further purified by semi-preparative HPLC on an Angilent Chemstation, using a reversed-phase column (Phenomenex, Luna Phenyl-Hexyl, 250 × 10 mm, 5 μm, 10 nm; 3.0 mL min^−1^, solvent A, H_2_O; solvent B, MeCN; eluted with constant 80% solvent B) to obtain compound **2** (8.3 mg). Fr.4 and Fr.5 were combined (3.7 g) and subjected to C18 reversed-phase MPLC (35 × 3 cm), eluting with a gradient of MeCN/H_2_O (45:55, 50:50, 60:40, 64:36, 66:34, 68:32, 70:30, 75:25, 80:20, 85:15, 90:10, 95:5 and 100:0, v/v, each of 600 mL) to yield 13 subfractions (Fr.4A–Fr.4M). Fr.6 (1.7 g) was purified by C18 reversed-phase MPLC (35 × 3 cm), eluting with a gradient of MeCN/H_2_O (40:60, 45:55, 55:45, 60:40, 63:37, 65:35, 70:30, 85:15, 95:5 and 100:0, v/v, each of 600 mL) to yield 10 subfractions (Fr.6A–Fr.6J). Fr.4M (250 mg) and Fr.6J (67 mg) were combined and subjected to Sephadex LH-20 CC eluted with CHCl_3_/MeOH (1:1), and the elution was combined with previous subfraction Fr.3I23 (49.0 mg) to get subfraction Fr.4M3. Fr.4M3 (93.0 mg) was further purified by semi-preparative HPLC on an Angilent Chemstation, using a reversed-phase column (Phenomenex, Gemini C18, 250 × 10 mm, 5 μm, 11 nm; 3.0 mL min^−1^, solvent A, H_2_O; solvent B, MeCN; eluted with constant 87% solvent B) to obtain compound **6** (7.4 mg) and **7** (47.0 mg).

Fr.4I (472.0 mg) was combined with Fr.3G (1.7 g) and subjected to C18 reversed-phase MPLC (30 × 3 cm), eluting with a gradient of MeCN/H_2_O (60:40, 70:30, 75:25, 80:20, 85:15, 85:15 and 90:0, v/v, each of 720 mL) to yield compound **3** (13.0 mg, subfraction Fr.3G2) and other 8 subfractions (Fr.3G1, Fr.3G3–Fr.3G9). Fr.3G7 (200.0 mg) was further purified by semi-preparative HPLC on a Hitachi Model D-2000 Elite station, using a reversed-phase column (Phenomenex, Gemini C18, 250 × 10 mm, 5 μm, 11 nm; 3.0 mL min^−1^, solvent A, H_2_O; solvent B, MeCN; eluted with constant 90% solvent B) to obtain compound **1** (8.0 mg) and **4** (113.0 mg). Fr.4K (62.0 mg) and Fr.3F (48.0 mg) were combined to Fr.6I (37.0 mg) and subjected to C18 reversed-phase MPLC (16 × 2 cm), eluting with a gradient of MeCN/H_2_O (60:40, 65:35, 70:30, 75:25, 80:20, 85:15, 90:10 and 100:0, v/v, each of 300 mL) to yield 8 subfractions (Fr.6I1–Fr.6I8). The subfraction Fr.6I6 (55.0 mg) was further purified by semi-preparative HPLC on an Angilent Chemstation, using a reversed-phase column (Phenomenex, Gemini C18, 250 × 10 mm, 5 μm, 11 nm; 3.0 mL min^−1^, solvent A, H_2_O; solvent B, MeCN; eluted with constant 70% solvent B) to obtain compound **8** (11.2 mg). Fr.4J (165.0 mg) was subjected to preparative HPLC on a Clarity station, using a reversed-phase column (YMC-Pack ODS-A, 250 × 20 mm, 5 μm, 12 nm; 10.0 mL min^−1^, solvent A, H_2_O; solvent B, MeCN; eluted with constant 75% solvent B) to give subfraction Fr.4J1 (12.0 mg). Fr.4J1 was subjected to Sephadex LH-20 CC eluted with CHCl_3_/MeOH (1:1), and further purified by PTLC and eluted with isooctane/EtOAc (1:1) to yield compound **5** (4.2 mg).

### 3.5. Characterization Data

4a-dechloronapyradiomycin A1 (**1**): pale yellow oil; 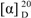 −51° (*c* 0.30, MeOH); UV (MeOH) λ_max_ (log ε): 202 (4.61), 257 (4.39), 312 (4.09), 362 (4.15) nm; IR ν_max_ 3294, 2920, 1701, 1616, 1373, 1261, 1145, 802 cm^−1^; ^1^H and ^13^C NMR data see Table 1; HRESIMS *m/z* 467.1595 [M + Na]^+^ (calcd for C_25_H_29_ClO_5_Na, 467.1596).

3-dechloro-3-brominapyradiomycin A1 (**2**): pale yellow oil; 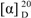 +23° (*c* 0.30, MeOH); UV (MeOH) λ_max_ (log ε): 202 (4.62), 253 (4.35), 297 (4.19), 364 (4.06) nm; IR ν_max_ 3348, 2939, 1701, 1612, 1450, 1369, 1253, 1072, 1018, 732 cm^−1^; ^1^H and ^13^C NMR data see Table 1; HRESIMS *m/z* 525.1047 [M + H]^+^ (calcd for C_25_H_31_ClBrO_5_, 525.1038).

3-chloro-6,8-dihydroxy-α-lapachone (**3**): orange powder; 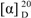 −22° (*c* 0.14, CHCl_3_); UV (MeOH) λ_max_ (log ε): 217 (4.42), 263 (4.19), 305 (3.95), 450 (3.42) nm; IR ν_max_ 3348, 2939, 1674, 1608, 1280, 1095, 1022, 887, 779 cm^−1^; ^1^H and ^13^C NMR data see Table 1; HRESIMS *m/z* 309.0532 [M + H]^+^ (calcd for C_15_H_14_ClO_5_, 309.0524).

### 3.6. Antibacterial, Cytotoxic and Antioxidative Assays

Minimal inhibition concentration (MIC) values for napyradiomycins **1**–**9** were determined against four bacterial strains (*Staphylococcus aureus* ATCC 29213, *Bacillus subtilis* SCSIO BS01, *Bacillus thuringensis* SCSIO BT01, *Escherichia coli* ATCC 25922) by previously described methods [40]. Cytotoxicities of all the nine compounds were assayed against SF-268, MCF-7, NCI-H460, and HepG-2 cell lines with the SRB method as previously described [41]. The antioxidant activity of napyradiomycins **1**–**9** was evaluated by DPPH (2,2-diphenyl-1-picryl-hydrazyl-hydrate) radical scavenging activity assays and compared with that of the reference antioxidant Trolox [27].

## 4. Conclusions

In this study, we discovered three new and six known napyradiomycins from the marine-derived *Streptomyces* sp. SCSIO 10428. Structure-activity relationship (SAR) studies revealed that (i) the oxidation at C-17 (e.g., compound **5**) had a significant negative effect on the antibacterial and cytotoxic activities, and (ii) the napyradiomycins with a linear 10-carbon monoterpenoid subunit at C-10a (e.g., compounds **2**, and **4**) and the napyradiomycins with a cyclized 6-membered ring at C-10a (e.g., compounds **6** and **7**) exhibited similar antibacterial and cytotoxic activities, while the napyradiomycin **9** with a 14-membered ring at C-10a had no antibacterial and cytotoxic activities. The new compound **2** exhibited comparable antibacterial activities to the well-known napyradiomycin A1 (**4**) and ampicillin. This study suggests that marine-derived actinomycetes are worthy of further exploration as novel drug candidates.

## Supplementary Files

Supplementary File 1 Supporting Information (PDF, 2484 KB)
