# Supplementary material for: Antibacterial and Cytotoxic New Napyradiomycins from the Marine-Derived Streptomyces sp. SCSIO 10428
Source: Mar Drugs. 2013 Jun 14;11(6):2113–25. doi: 10.3390/md11062113 (PMC3721223; doi:10.3390/md11062113)
Supplement: Supplementary File 1 — Supporting Information (PDF, 2484 KB) [file marinedrugs-11-02113-s001.pdf]

Supporting Information

Table of Content

Elucidation of known compounds **4**–**9**	2–3
**Table S1.** NMR data of napyradiomycin A1 (**4**)	4
**Figure S1.** Phylogenetic dendrogram of the strain *Streptomyces* sp. SCSIO 10428	5
**Figure S2.** Spectral data for compound **1**	6–11
(**A**) HRESIMS, IR, and UV spectra of compound **1**	6
(**B**) The ^{1}H NMR spectrum of compound **1** measured in $CDCl_3$	7
(**C**) The ^{13}C and the DEPT 135 NMR spectra of compound **1** measured in $CDCl_3$	8
(**D**) The HSQC spectrum of compound **1** measured in $CDCl_3$	9
(**E**) The HMBC spectrum of compound **1** measured in $CDCl_3$	10
(**F**) The COSY spectrum of compound **1** measured in $CDCl_3$	11
**Figure S3.** Spectral data for compound **2**	12–17
(**A**) HRESIMS, IR, and UV spectra of compound **2**	12
(**B**) The ^{1}H NMR spectrum of compound **2** measured in $CDCl_3$	13
(**C**) The ^{13}C and the DEPT 135 NMR spectra of compound **2** measured in $CDCl_3$	14
(**D**) The HSQC spectrum of compound **2** measured in $CDCl_3$	15
(**E**) The HMBC spectrum of compound **2** measured in $CDCl_3$	16
(**F**) The COSY spectrum of compound **2** measured in $CDCl_3$	17
**Figure S4.** Spectral data for compound **3**. (NMR measured in CD_3OD)	18–23
(**A**) HRESIMS, IR, and UV spectra of compound **3**	18
(**B**) The ^{1}H NMR spectrum of compound **3** measured in CD_3OD	19
(**C**) The ^{13}C and the DEPT 135 NMR spectra of compound **3** measured in CD_3OD	20
(**D**) The HSQC spectrum of compound **3** measured in CD_3OD	21
(**E**) The HMBC spectrum of compound **3** measured in CD_3OD	22
(**F**) The COSY spectrum of compound **3** measured in CD_3OD	23
**Figure S5.** Spectral data for compound **3**. (NMR measured in $CDCl_3$)	24–26
(**A**) The ^{1}H NMR spectrum of compound **3** measured in $CDCl_3$	24
(**B**) The ^{13}C and the DEPT 135 NMR spectra of compound **3** measured in $CDCl_3$	25
(**C**) The HMBC spectrum of compound **3** measured in $CDCl_3$	26

Elucidation of Known Compounds 4–9

Compound **4** was obtained as pale yellow oils. The UV absorption pattern was observed at 253, 298 and 364 nm. Its sodium D line rotation was $[\alpha]_D^{20}$ +51 ° (*c* 0.30, MeOH). The molecular formula was determined as $C_{25}H_{30}Cl_2O_5$ by HRESIMS (*m/z* 481.1555 $[M + H]^+$, calcd 481.1549). The IR spectrum of **4** showed broad absorptions for multiple hydroxyl groups band around 3367 cm^{-1}, and also 1689 cm^{-1} characteristic for the conjugated carbonyl groups functionality. The 1D and 2D NMR data of **4** was shown in Table S1. Comparing these spectroscopic data with the literature [1,2], we found that **4** was almost identical to napyradiomycin A1. Thus **4** was identified as napyradiomycin A1.

Compound **5** was obtained as pale yellow oils. The molecular formula was determined as $C_{25}H_{28}Cl_2O_6$ by HRESIMS (*m/z* 495.1333 $[M + H]^+$, calcd 495.1336). ^1H NMR ($CDCl_3$, 500 MHz) δ_H 11.87 (1H, s), 9.39 (1H, s), 7.10 (1H, d, *J* = 2.4), 6.71 (1H, d, *J* = 2.4), 6.38 (1H, t, *J* = 5.0), 4.85 (1H, t, *J* = 10.0), 4.43 (1H, dd, *J* = 12.0, 4.0), 2.70 (1H, dd, *J* = 14.0, 7.5), 2.56 (1H, dd, *J* = 14.0, 7.5), 2.46 (1H, m), 2.40 (1H, m), 2.15 (2H, m), 1.93 (2H, m), 1.67 (3H, s), 1.49 (3H, s), 1.37 (3H, s), 1.19 (3H, s). ^{13}C and DEPT 135 NMR ($CDCl_3$, 125 MHz) δ_C 196.7 (s), 194.7 (s), 194.0 (s), 165.1 (s), 164.6, (s), 155.2 (d), 139.6 (s), 139.5 (s), 135.2 (s), 117.1 (d), 109.5 (s), 109.4 (d), 107.9 (d), 83.7 (s), 79.4 (s), 78.9 (s), 58.8 (d), 42.7 (t), 40.6 (t), 37.8 (t), 29.0 (q), 26.8 (t), 22.3 (q), 16.3 (q), 9.1 (q). The ^1H and ^{13}C NMR of compound **5** were identical to those of 18-oxonapyradiomycin A1 in literature [3]. Thus **5** was identified as 18-oxonapyradiomycin A1.

Compound **6** was obtained as pale yellow oils. The molecular formula was determined as $C_{25}H_{29}Cl_3O_5$ by HRESIMS (*m/z* 515.1183 $[M + H]^+$, calcd 515.1159). ^1H NMR ($CDCl_3$, 500 MHz) δ_H 12.04 (1H, br s), 7.16 (1H, br s), 6.74 (1H, br s), 4,78 (1H, s), 4.75 (1H, s), 4.44 (1H, dd, *J* = 12.0, 3.5), 3.78 (1H, dd, *J* = 11.5, 4.0), 2.64 (1H, dd, *J* = 15.0, 8.0), 2.52 (1H, dd, *J* = 13.0, 4.0), 2.35 (1H, t, *J* = 12.5), 2.25 (1H, dt, *J* = 13.0, 9.5), 2.03 (1H, m), 1.99 (1H, br d, *J* = 8.5), 1.93 (1H, ddd, *J* = 12.5, 3.5), 1.72 (1H,m), 1.61 (1H, br d, *J* = 15.5), 1.38 (3H, s), 1.19 (3H, s), 0.70 (3H, s), 0.58 (3H, s). ^{13}C NMR and DEPT 135 ($CDCl_3$, 125 MHz) δ_C 194.0 (s), 193.5 (s), 165.5 (s), 164.1 (s), 145.4 (s), 135.1 (s), 110.3 (t), 109.5 (d), 108.6 (s), 108.4 (d), 84.3 (s), 80.9 (s), 78.8 (s), 70.8 (d), 58.8 (d), 45.9 (d), 42.8 (t), 41.8 (s), 35.6 (t), 35.0 (t), 34.6 (t), 29.0 (q), 26.4 (q), 22.4 (q), 15.5 (q). The ^1H and ^{13}C NMR of compound **6** were identical to those of napyradiomycin B1 in literature [1,2]. Thus **6** was identified as napyradiomycin B1.

Compound **7** was obtained as pale yellow oils. The molecular formula was determined as $C_{25}H_{29}BrCl_2O_5$ by HRESIMS (*m/z* 559.0662 $[M + H]^+$, calcd 559.0654). ^1H NMR ($CDCl_3$, 500 MHz) δ_H 12.03 (1H, br s), 7.15 (1H, br s), 6.74 (1H, br s), 4,78 (1H, s), 4.76 (1H, s), 4.45 (1H, dd, *J* = 12.0, 4.0), 4.03 (1H, dd, *J* = 11.0, 4.0), 2.66 (1H, dd, *J* = 15.0, 8.5), 2.53 (1H, dd, *J* = 14.0, 4.0), 2.35 (1H, dd, *J* = 12.0, 4.0), 2.21 (1H, m), 2.19 (1H, m), 2.04 (1H, br d, *J* = 8.0), 1.93 (1H, m), 1.92 (1H,m), 1.63 (1H, br d, *J* = 15.0), 1.38 (3H, s), 1.20 (3H, s), 0.73 (3H, s), 0.63 (3H, s). ^{13}C and DEPT 135 NMR ($CDCl_3$, 125 MHz) δ_C 194.0 (s), 193.5 (s), 165.5 (s), 164.2 (s), 145.3 (s), 135.1 (s), 110.2 (t), 109.5 (d), 108.6 (s), 108.5 (d), 84.3 (s), 80.9 (s), 78.8 (s), 66.7 (d), 58.8 (d), 45.8 (d), 42.8 (t), 41.8 (s), 37.3 (t), 36.0 (t), 35.4 (t), 29.0 (q), 27.8 (q), 22.4 (q), 16.4 (q). The ^1H and ^{13}C NMR of compound **7** were identical to those of napyradiomycin B3 in literature [1,2]. Thus compound **7** was identified as napyradiomycin B3.

Compound **8** was obtained as pale yellow oils. The molecular formula was determined as $C_{25}H_{31}ClO_5$ by HRESIMS (*m/z* 469.1757 $[M + Na]^+$, calcd 469.1752). ^1H NMR ($CDCl_3$, 500 MHz) δ_H 11.97 (1H, br s), 7.04 (1H, s), 6.71 (1H, s), 5.01 (1H, dd, *J* = 8.0, 8.0), 4.92 (1H, dd, *J* = 8.0, 8.0), 4.82 (1H, dd, *J* = 8.0, 8.0), 3.00 (1H, m), 2.96 (1H, m), 2.47 (1H, br dd, *J* = 15.0, 8.0), 2.29 (1H, dd, *J* = 13.5, 8.0), 1.95 (1H, m), 1.89 (1H, m), 1.69 (3H, s), 1.58 (3H, s), 1.55 (3H, s), 1.29 (3H, s), 1.28 (3H, s). ^{13}C NMR and DEPT 135 (CD_3OD, 125 MHz) δ_C 197.5 (s), 196.5 (s), 167.4 (s), 166.0 (s), 141.0 (s), 138.5 (s), 136.8 (s), 132.5 (s), 125.1 (d), 118.3 (d), 117.8 (d), 111.0 (s), 109.1 (d), 108.5 (d), 86.0 (s), 85.1 (s), 40.9 (t), 39.6 (t), 39.7 (t), 27.5 (t), 25.9 (q), 25.9 (q), 17.8 (q), 17.7 (q), 16.2 (q). The ^1H and ^{13}C NMR of compound **8** were identical to those of naphthomevalin in literature [4]. Thus compound **8** was identified as naphthomevalin.

Compound **9** was obtained as pale yellow oils. The molecular formula was determined as $C_{25}H_{26}Cl_2O_5$ by HRESIMS (*m/z* 477.1238 $[M + H]^+$, calcd 477.1230). ^1H NMR ($CDCl_3$, 500 MHz) δ_H 12.23 (1H, s), 6.89 (1H, s), 5.15 (1H, dd, *J* = 11.5, 3.5), 4.59 (2H, s), 4.47 (1H, m), 4.46 (1H, m), 3.74 (1H, d, *J* = 12.0), 2.91 (1H, d, *J* = 12.0), 2.65 (1H, dd, *J* = 14.5, 4.0), 2.48 (2H, m), 2.47 (1H, m), 2.12 (1H, m), 2.01 (1H, m), 1.86 (1H, m), 1.52 (3H, s), 1.37 (3H, s), 1.14 (3H, s). ^{13}C and DEPT 135 NMR ($CDCl_3$, 125 MHz) δ_C 196.4 (s), 194.5 (s), 164.8 (s), 157.9 (s), 138.0 (s), 134.1 (s), 133.7 (s), 132.1 (s), 131.4 (s), 116.7 (d), 113.7 (d), 112.4 (s), 85.7 (s), 79.3 (s), 76.3 (s), 69.0 (t), 58.7 (d), 43.2 (t), 42.0 (t), 39.6 (t), 31.8 (t), 29.4 (q), 24.7 (t), 22.0 (q), 14.8 (q). The ^1H and ^{13}C NMR of compound **9** were identical to those of napyradiomycin SR in literature [3]. Thus compound **9** was identified as napyradiomycin SR.

Table S1. NMR data of napyradiomycin A1 (**4**) (measured in $CDCl_3$, at 500 MHz for ^1H and 125 MHz for ^{13}C with reference to the solvent signals, δ in ppm).

No.	δ_C	δ_H (*J* in Hz)	HMBC	COSY
2	79.0 s			
2-CH_3	29.0 q	1.50 s	2, 3	
2-CH_3	22.4 q	1.18 s	2, 3, 2-CH_3	
3	59.0 d	4.43 dd (4.5, 11.5)	2	4
4	42.9 t	2.48 dd (4.5, 14.0)<br>2.41 dd (11.5, 14.0)	2, 3, 4a, 10	3
4a	79.2 s			
5	194.0 s			
5a	110.5 s			
6	165.0 s			
6-OH		11.84 s	5a, 6, 7	
7	109.7 d	6.73 d (2.5)	5a, 6, 9	
8	163.7 s			
9	107.9 d	7.23 d (2.5)	5a, 10	
9a	135.6 s			
10	196.3 s			
10a	83.8 s			
11	41.5 t	2.70 br d (8.0)	4a, 10, 10a, 12, 13	12
12	115.1 d	4.71 br t (8.0)	11, 13-CH_3	11
13	143.0 s			
13-CH_3	16.6 q	1.32 s	12, 13, 14	
14	39.9 t	1.60 m	15, 16, 17	
15	26.2 t	1.60 m		16
16	123.9 d	4.89 br s		15
17	131.9 s			
17-CH_3	17.7 q	1.50 s	16, 17, 17-CH_3	
17-CH_3	25.8 q	1.62 s	17-CH_3	

Figure S1. Phylogenetic dendrogram of the strain *Streptomyces* sp. SCSIO 10428 and its closest relatives reconstructed by the neighbor-joining method based on 16S rDNA gene sequences.

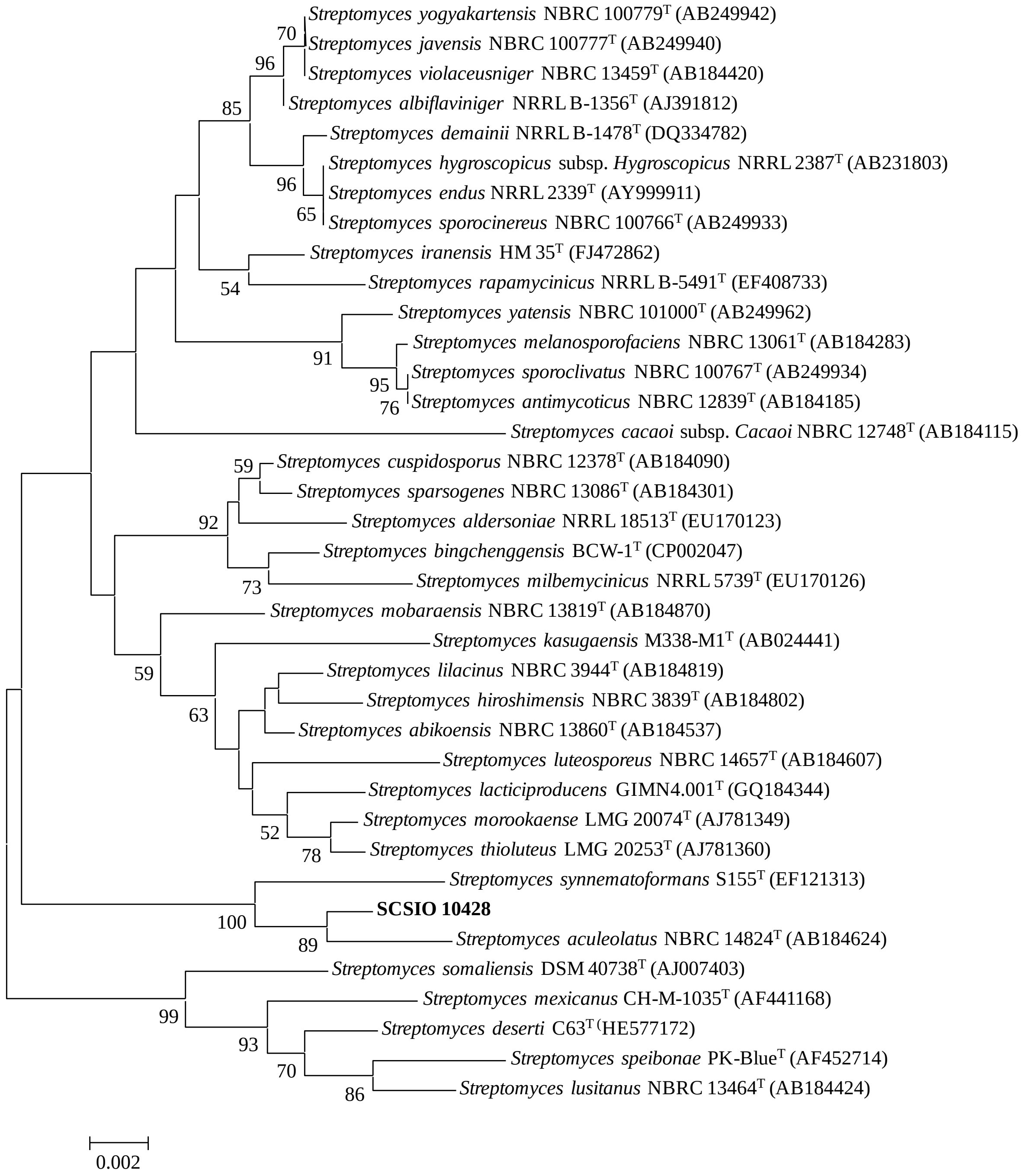

Figure S2. Spectral data for compound **1**. (**A**) HRESIMS (**a**), IR (**b**) and UV (**c**) spectra of compound **1**.

(**a**) HRESIMS spectrum of compound **1**.

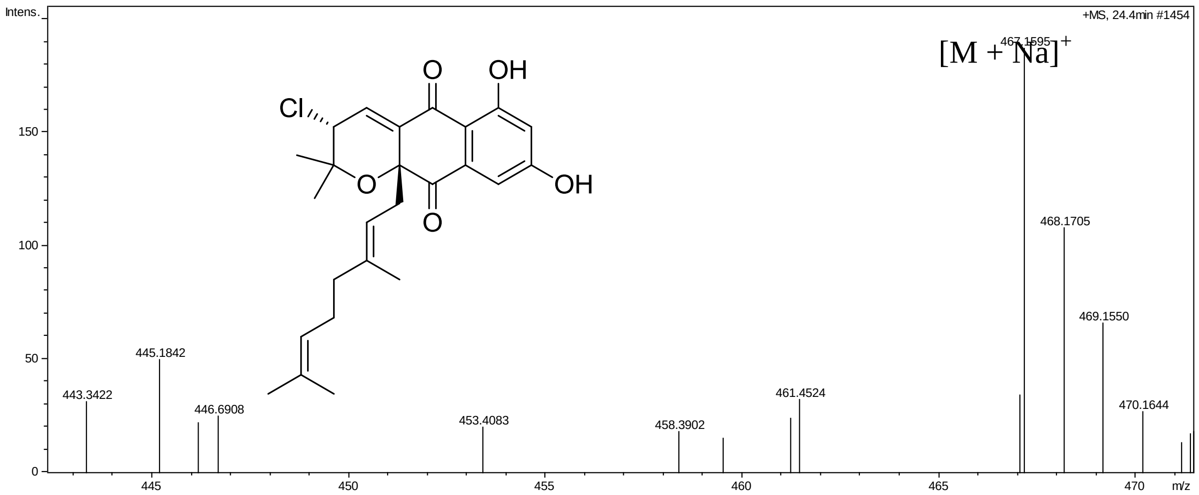

(**b**) IR spectrum of compound **1**.

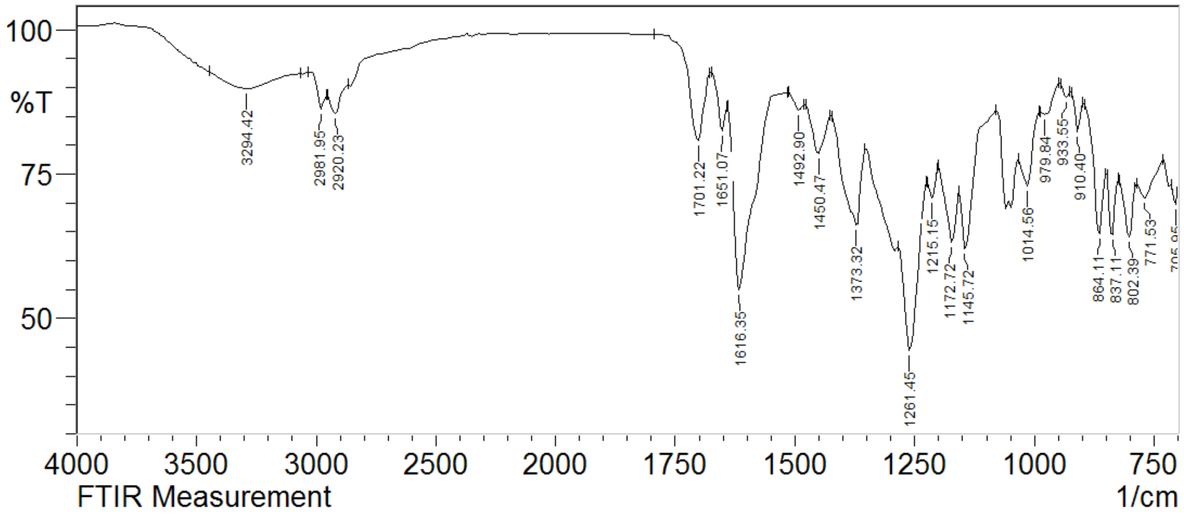

(**c**) The UV spectrum of compound **1**.

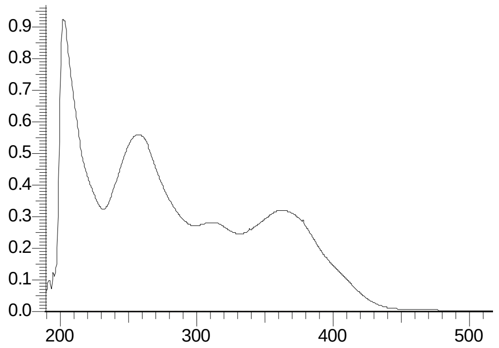

Figure S2. (**B**) The ^{1}H NMR spectrum of compound **1** measured in $CDCl_3$.

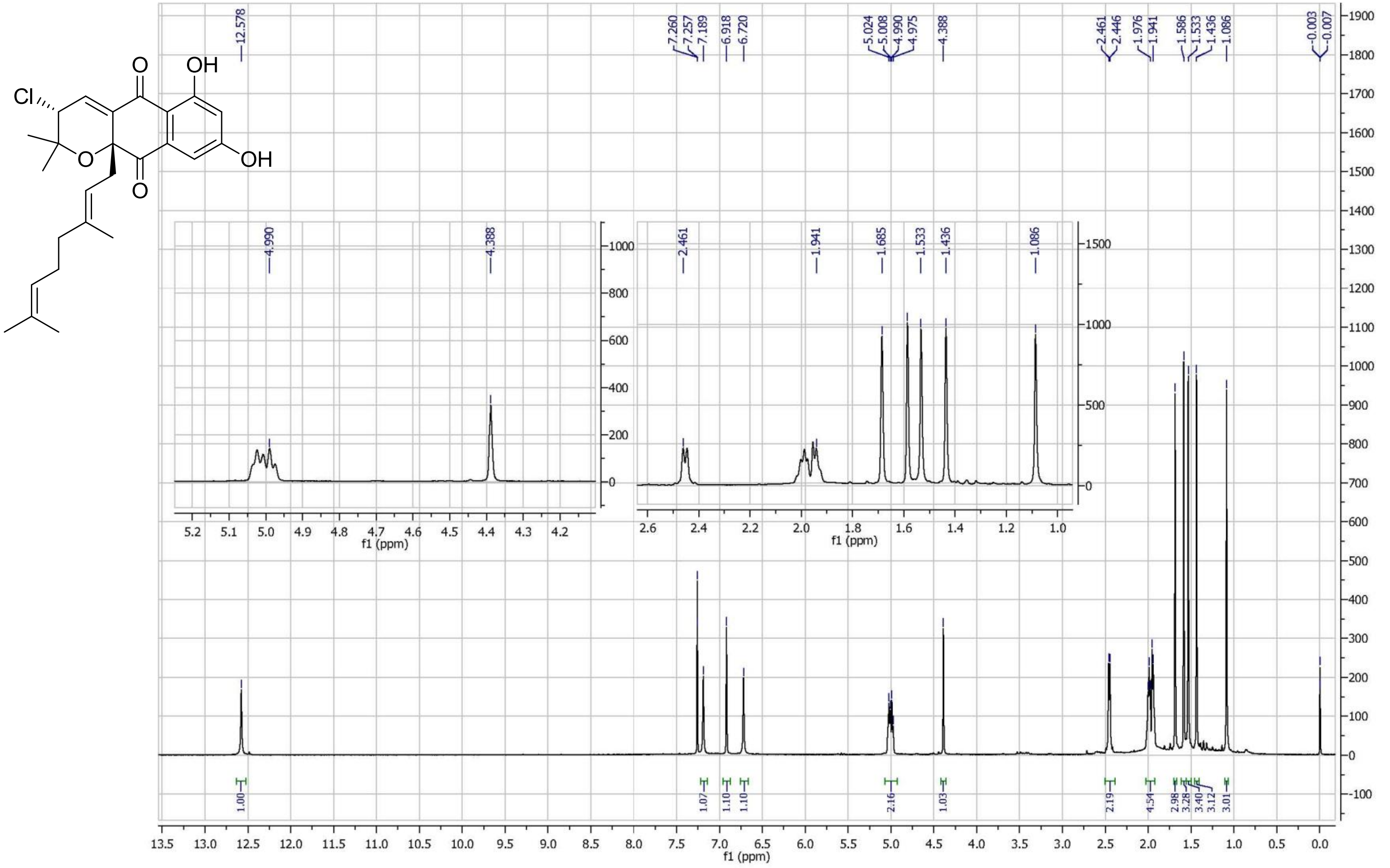

Figure S2. (**C**) The ^{13}C and the DEPT 135 NMR spectra of compound **1** measured in $CDCl_3$.

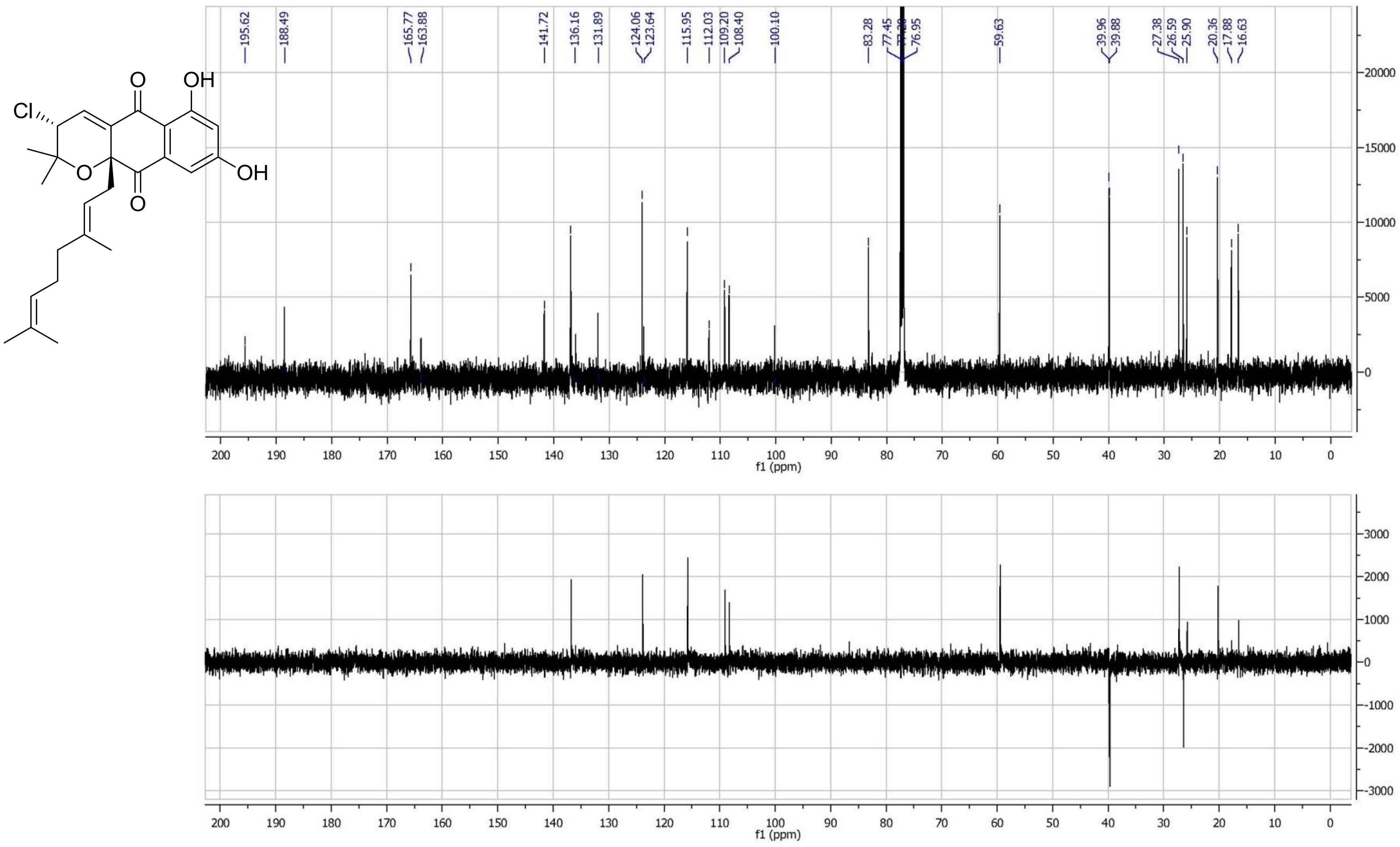

Figure S2. (**D**) The HSQC spectrum of compound **1** measured in $CDCl_3$.

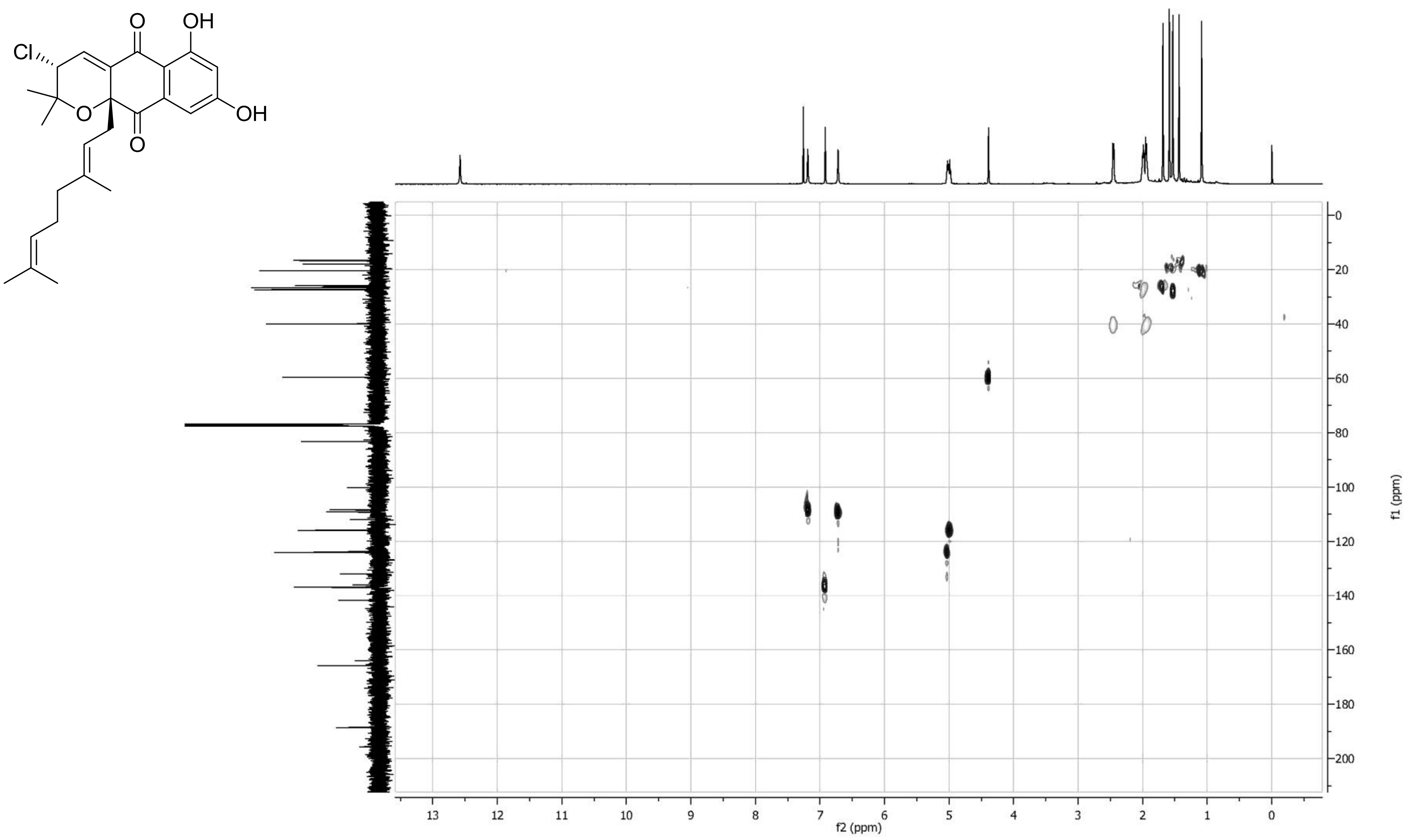

Figure S2. (**E**) The HMBC spectrum of compound **1** measured in $CDCl_3$.

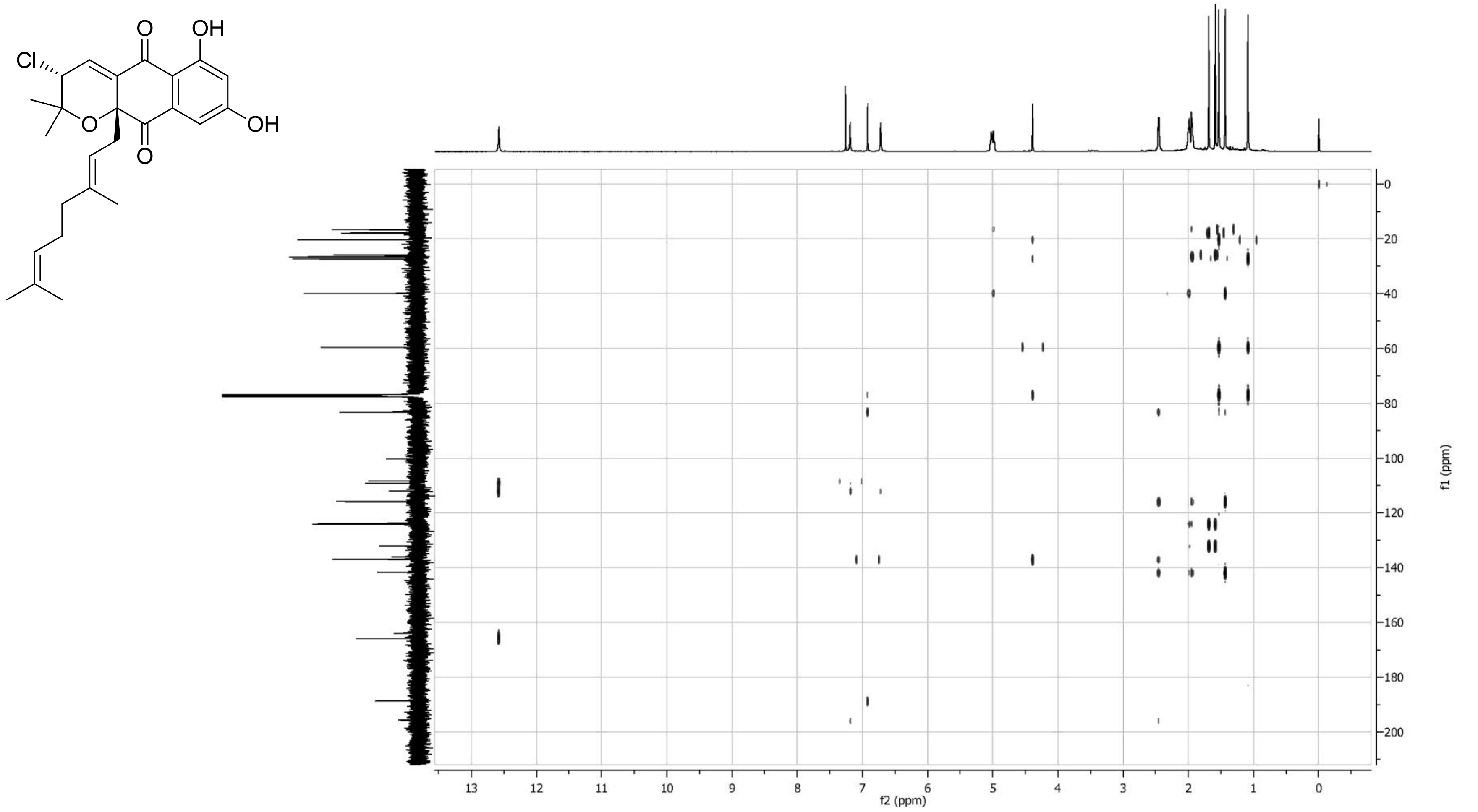

Figure S2. (**F**) The COSY spectrum of compound **1** measured in $CDCl_3$.

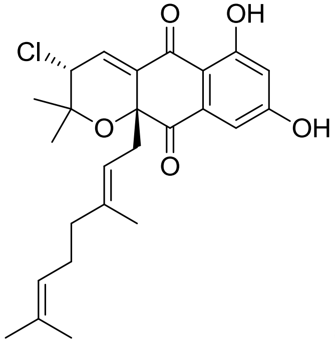

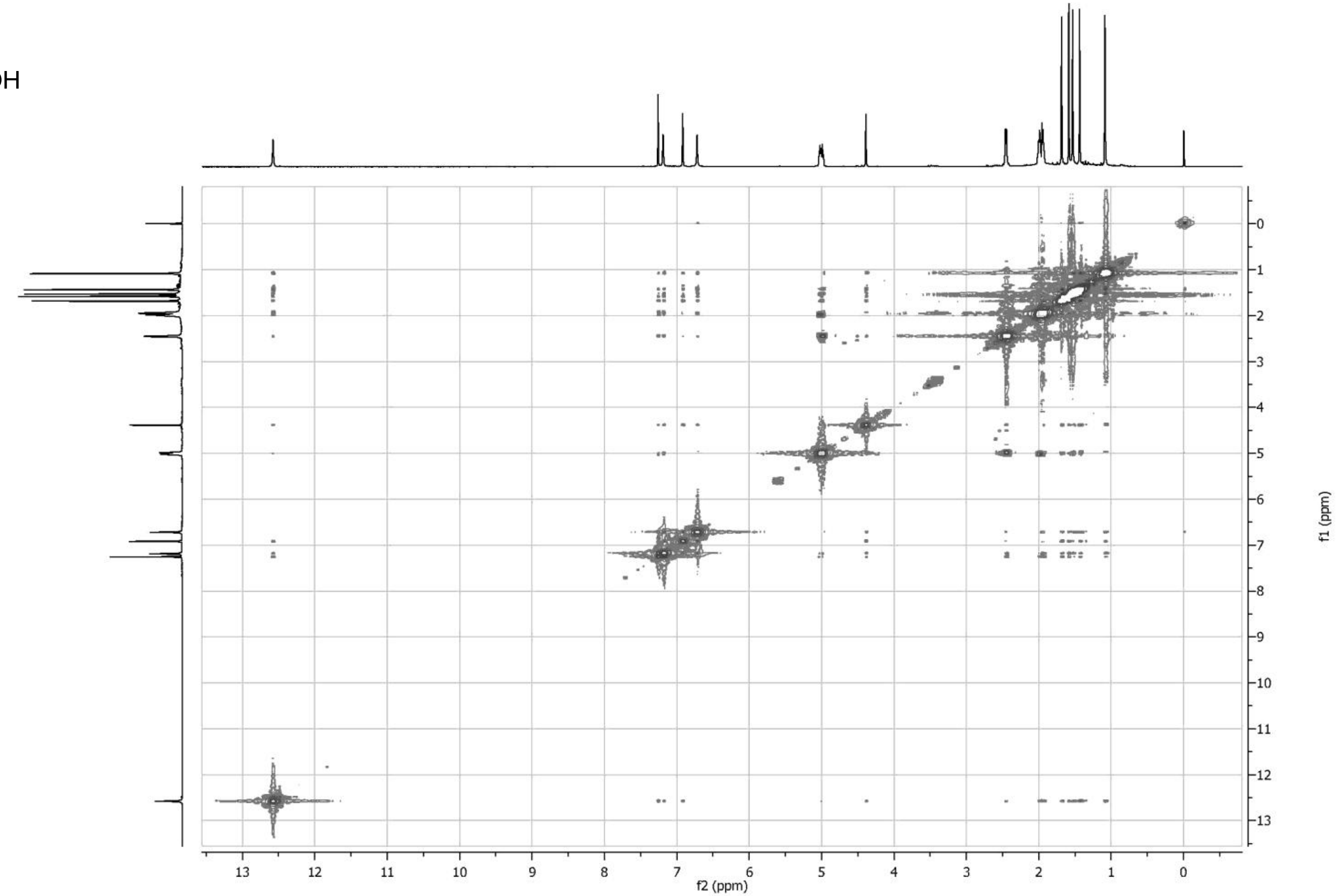

Figure S3. Spectral data for compound **2**. (**A**) HRESIMS (**a**), IR (**b**) and UV (**c**) spectra of compound **2**.

(**a**) HRESIMS spectrum of compound **2**.

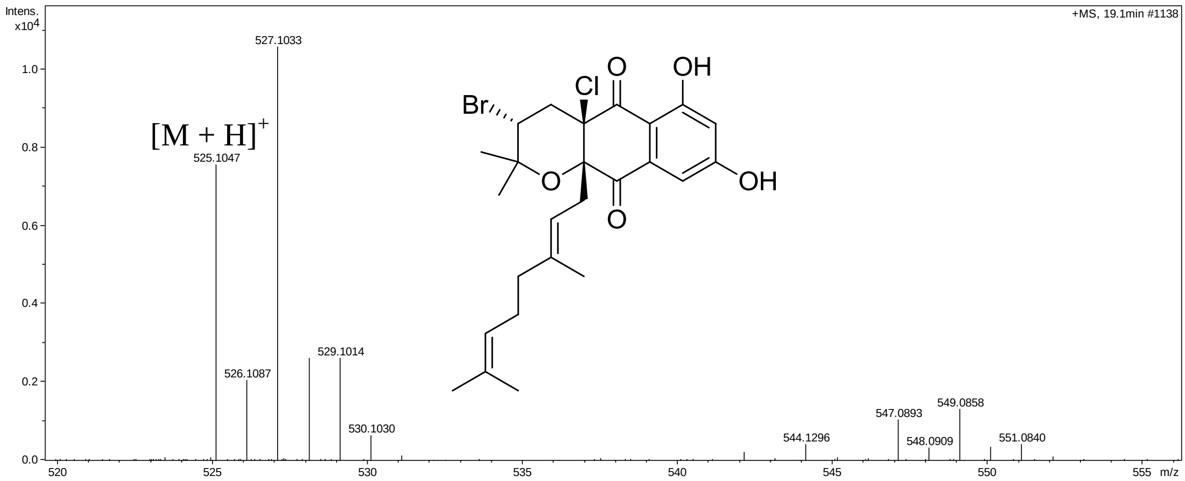

(**b**). IR spectrum of compound **2**.

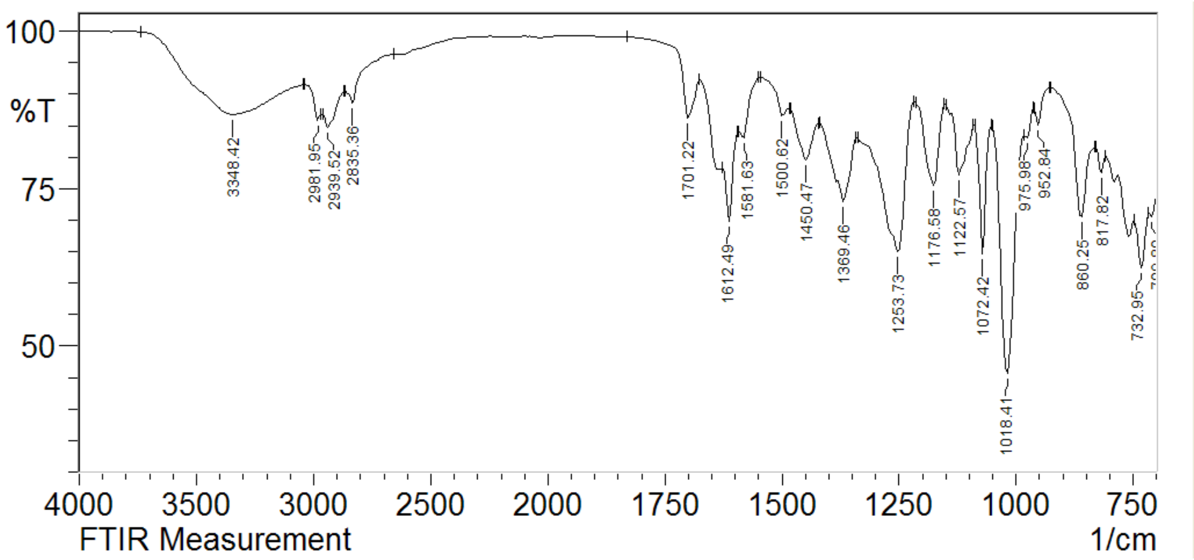

(**c**) UV spectrum of compound **2**.

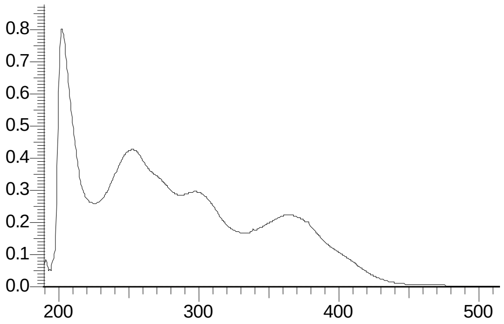

Figure S3. (**B**) The ^{1}H NMR spectrum of compound **2** measured in $CDCl_3$.

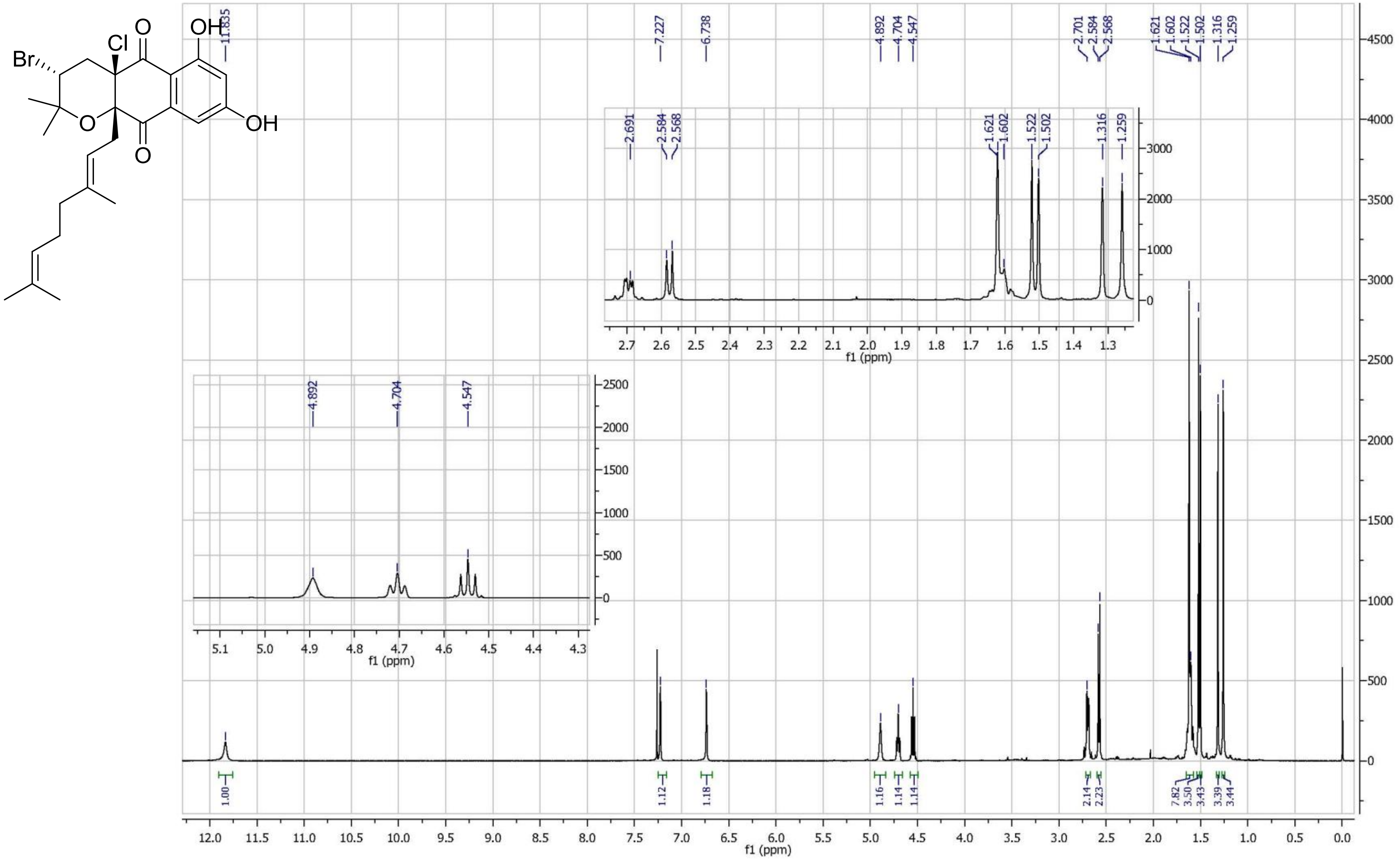

Figure S3. (**C**) The ^{13}C and the DEPT 135 NMR spectra of compound **2** measured in $CDCl_3$.

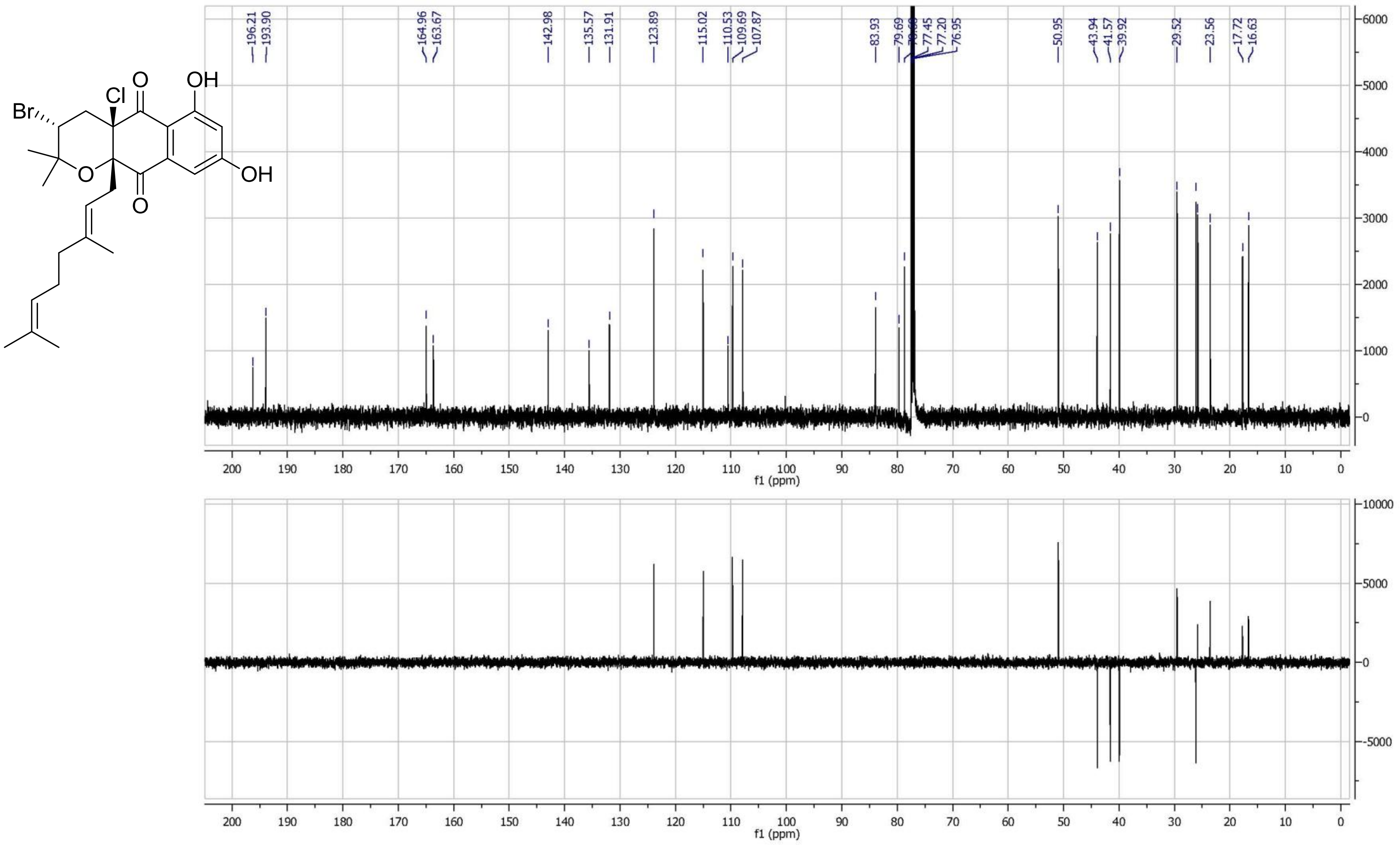

Figure S3. (**D**) The HSQC spectrum of compound **2** measured in $CDCl_3$.

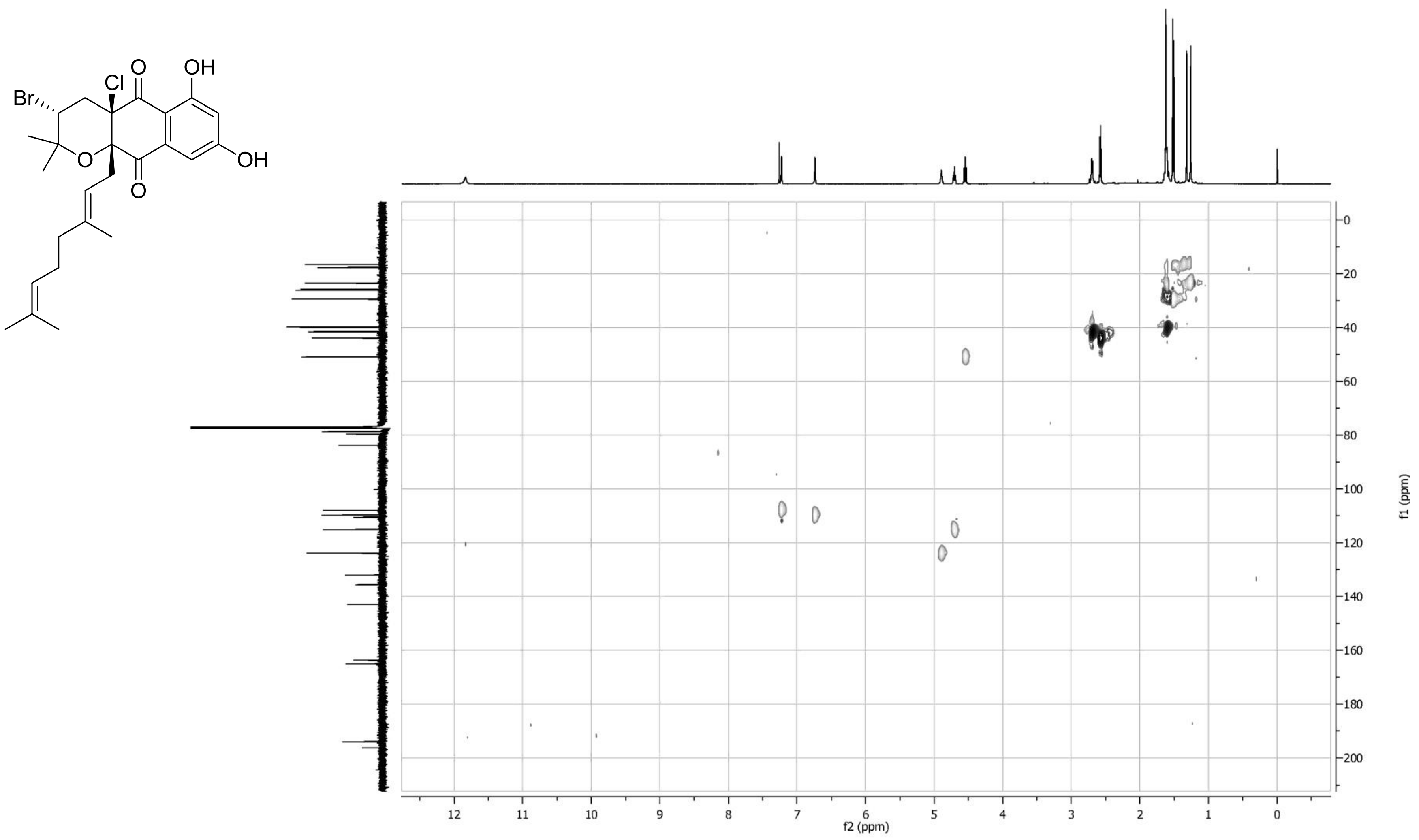

Figure S3. (**E**) The HMBC spectrum of compound **2** measured in $CDCl_3$.

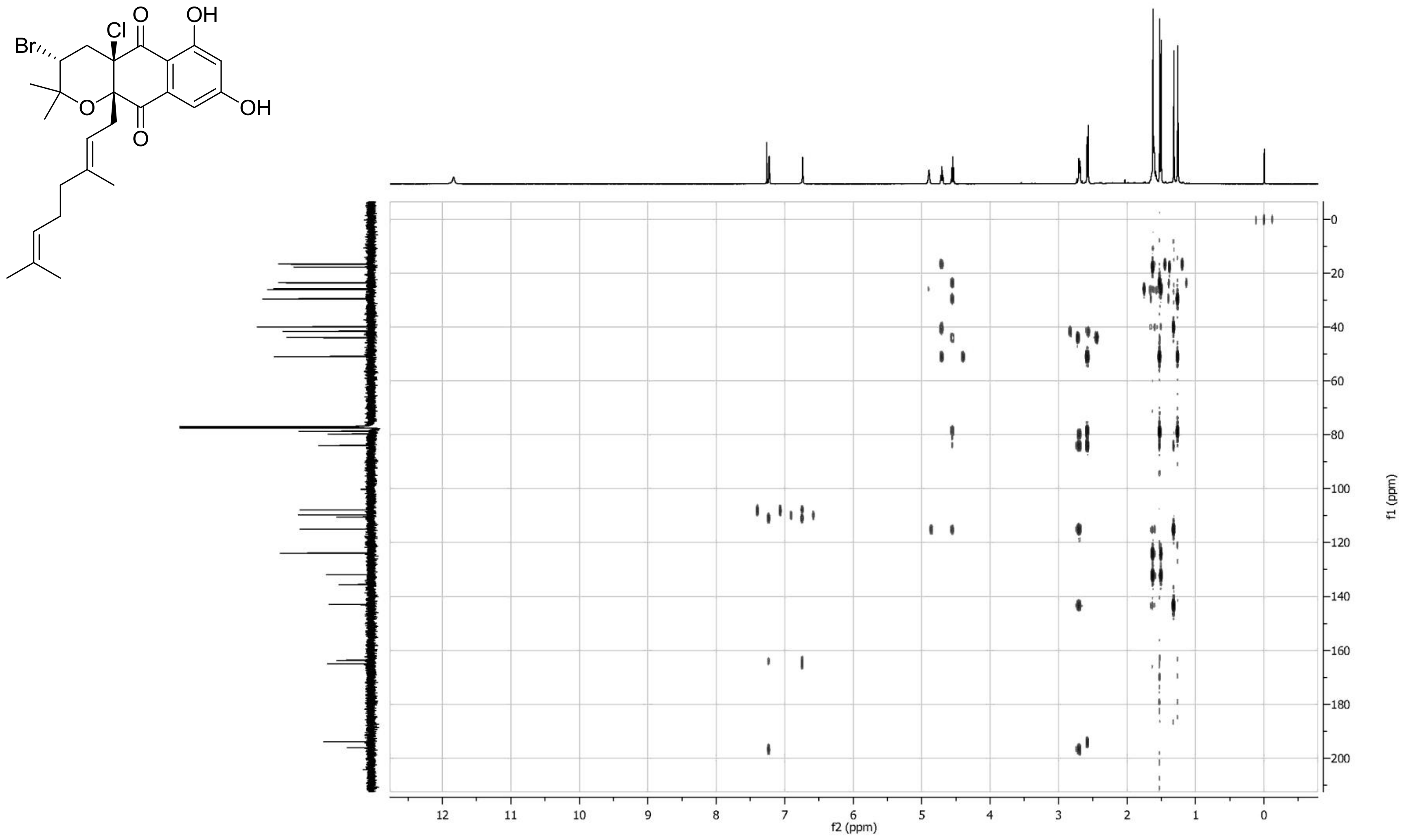

Figure S3. (**F**) The COSY spectrum of compound **2** measured in $CDCl_3$.

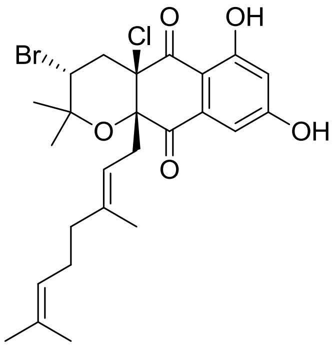

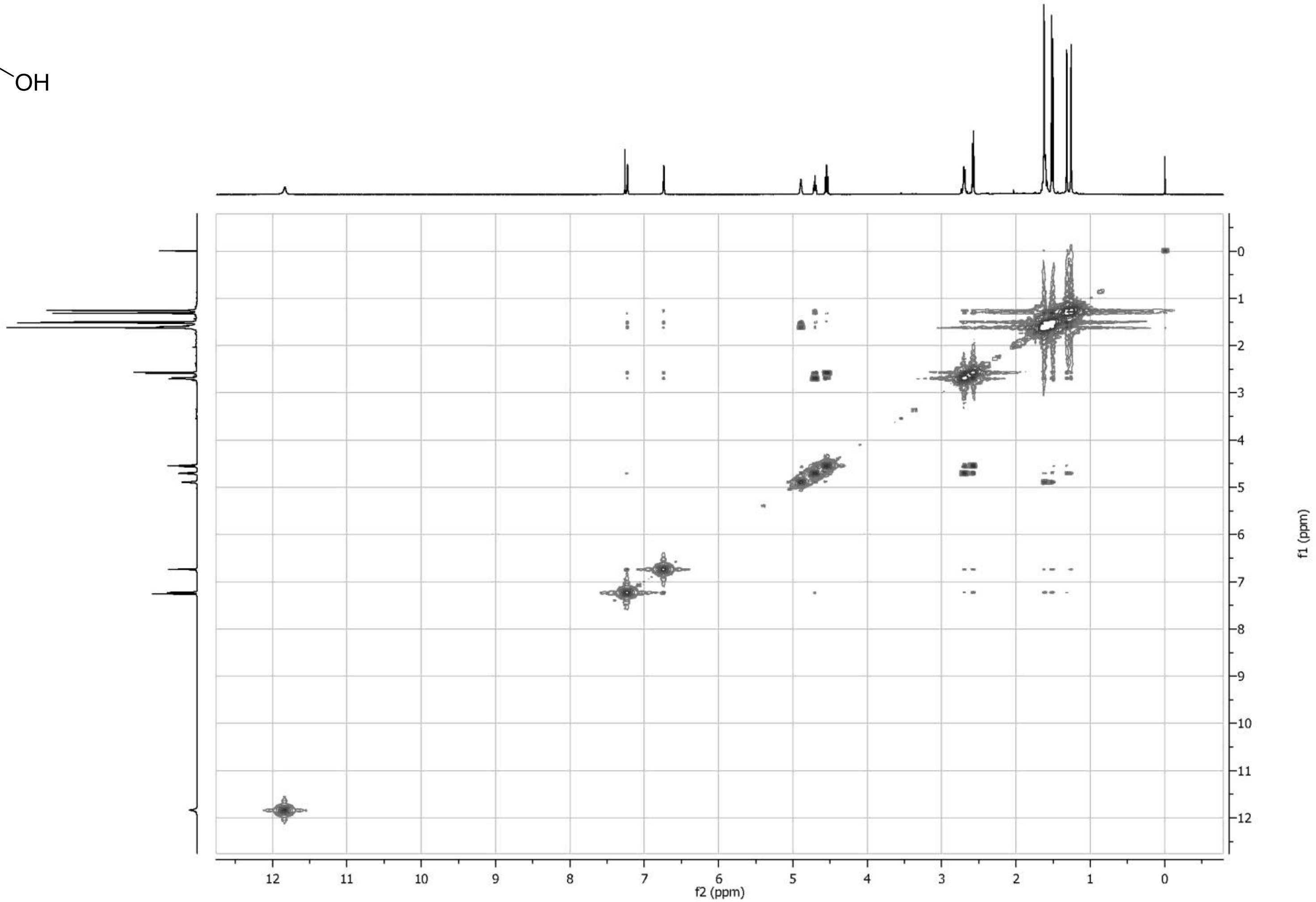

Figure S4. Spectral data for compound **3**. (**A**) HRESIMS (**a**), IR (**b**) and UV (**c**) spectra of compound **3**.

(**a**). HRESIMS spectrum of compound **3**.

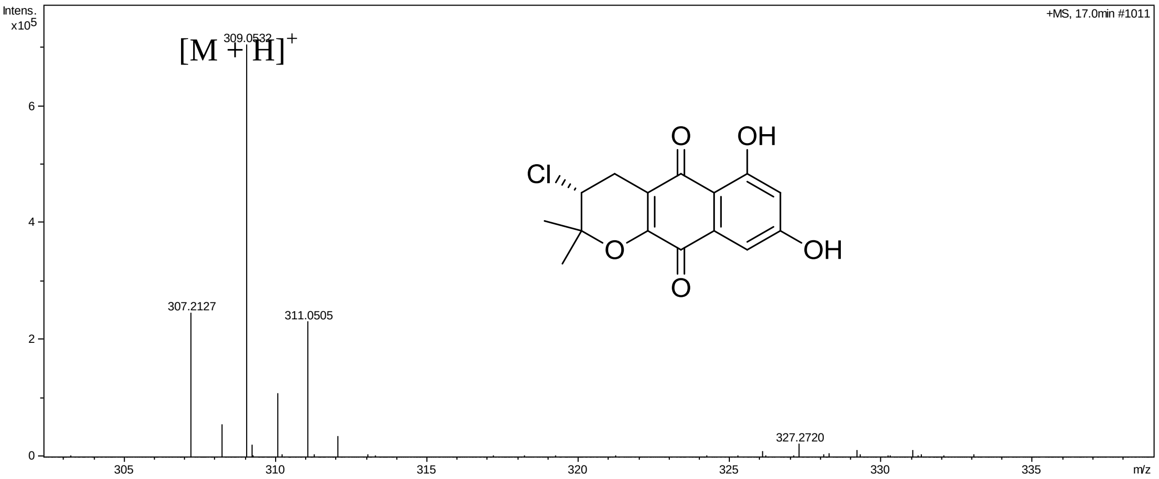

(**b**). IR spectrum of compound **3**.

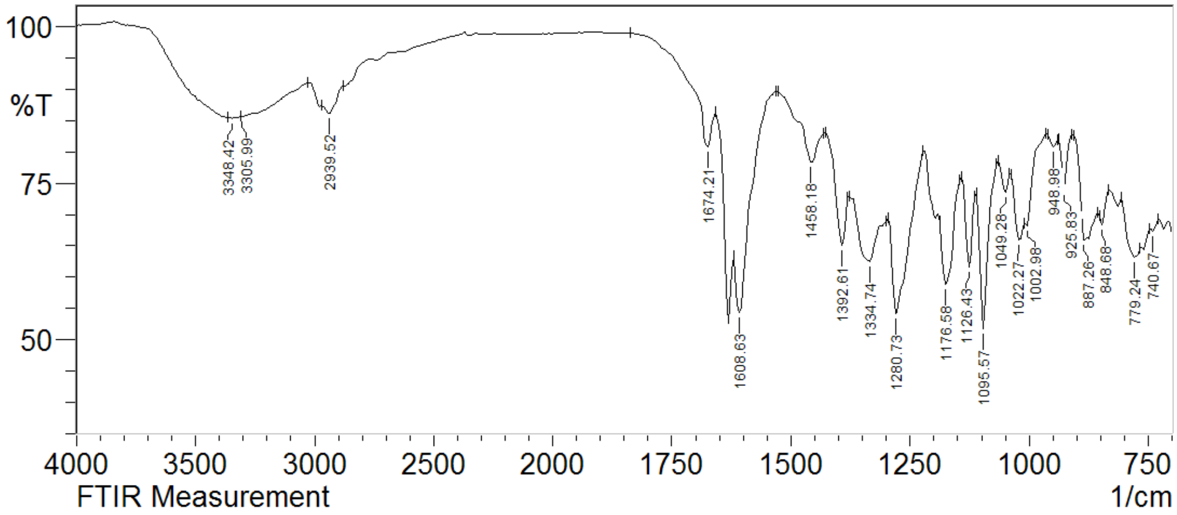

(**c**). UV spectrum of compound **3**.

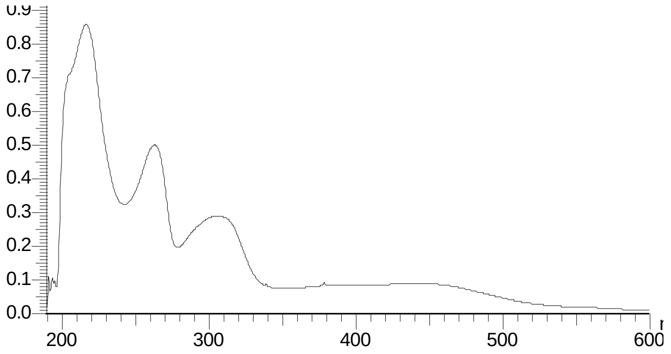

Figure S4. (**B**) The ^{1}H NMR spectrum of compound **3** measured in CD_3OD.

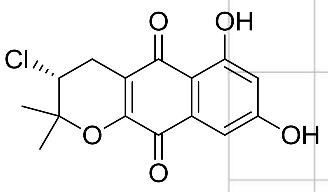

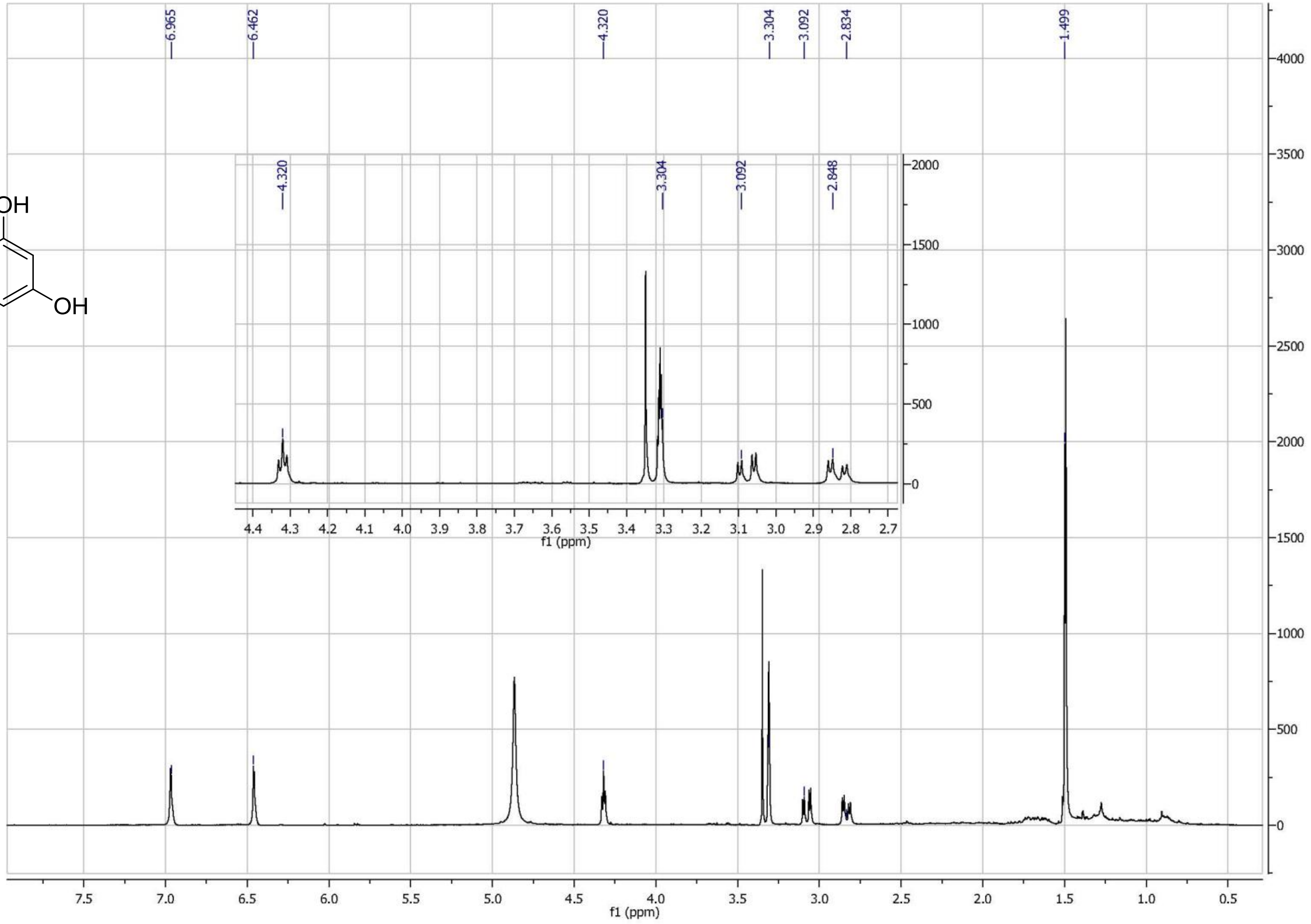

Figure S4. (**C**) The ^{13}C and the DEPT 135 NMR spectrum of compound **3** measured in CD_3OD.

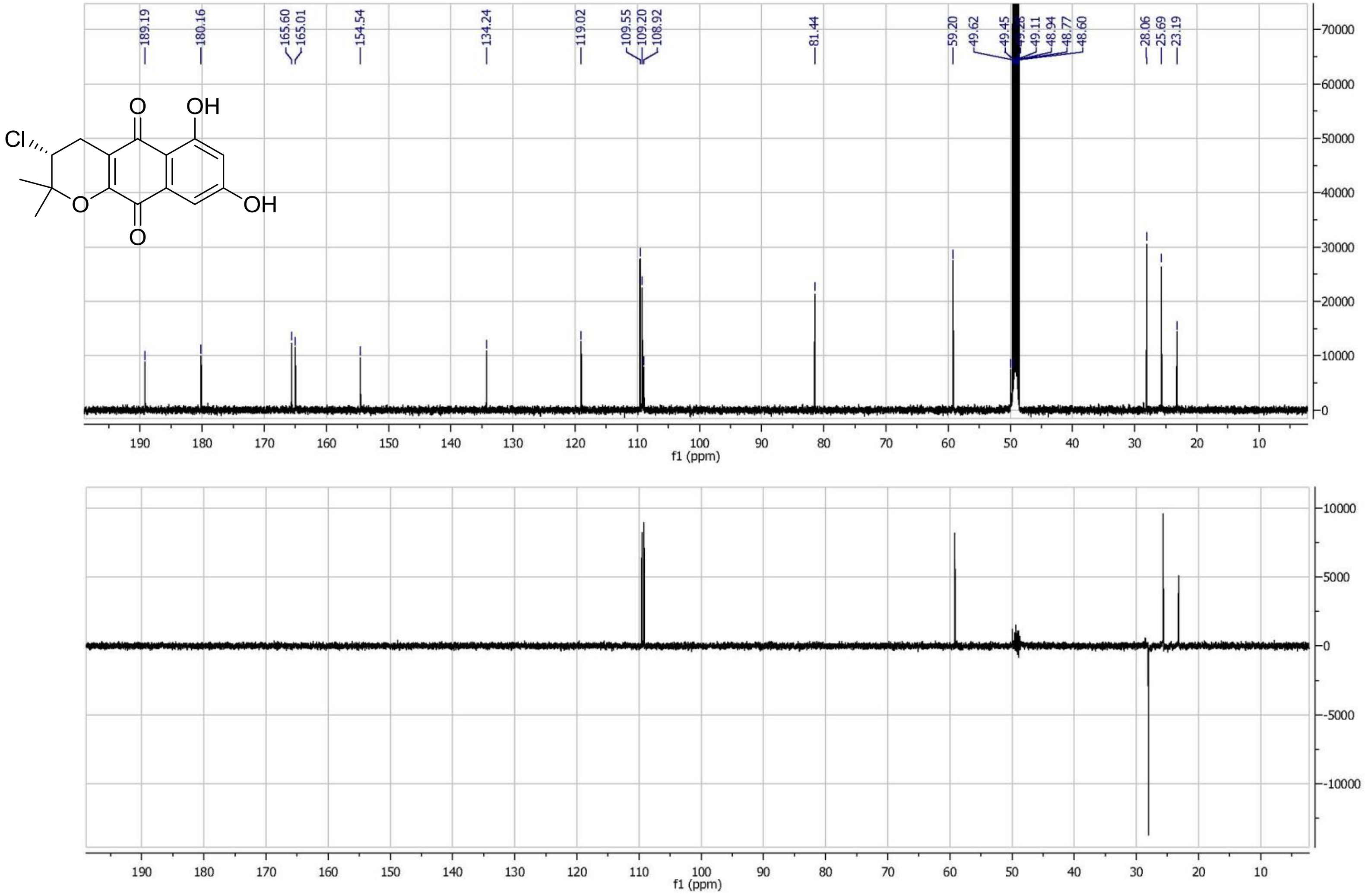

Figure S4. (**D**) The HSQC spectrum of compound **3** measured in CD_3OD.

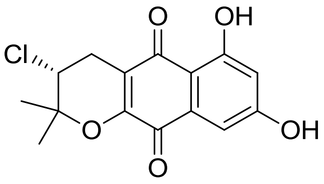

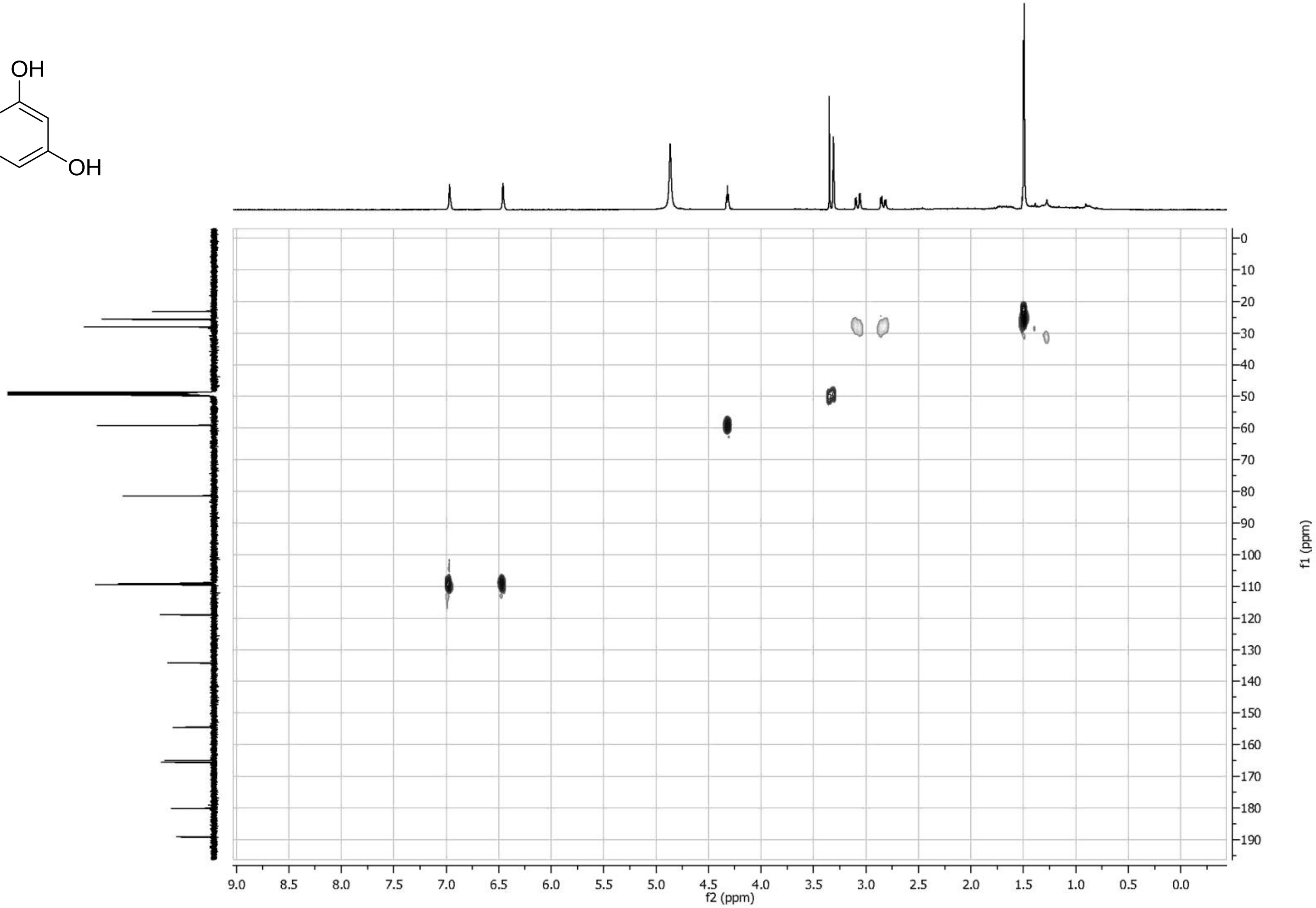

Figure S4. (**E**) The HMBC spectrum of compound **3** measured in CD_3OD.

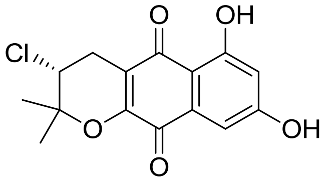

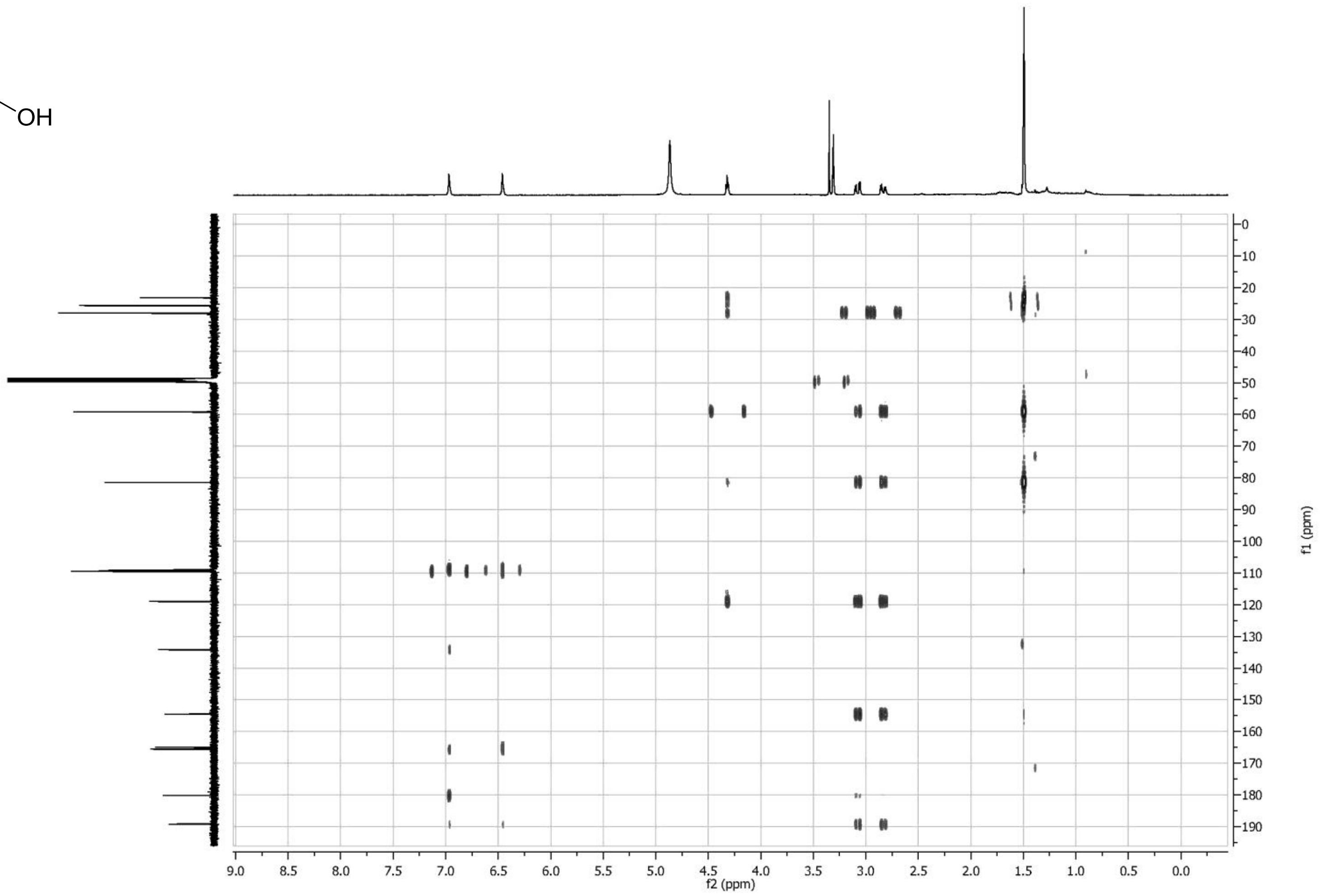

)

Figure S4. (**F**) The COSY spectrum of compound **3** measured in CD_3OD.

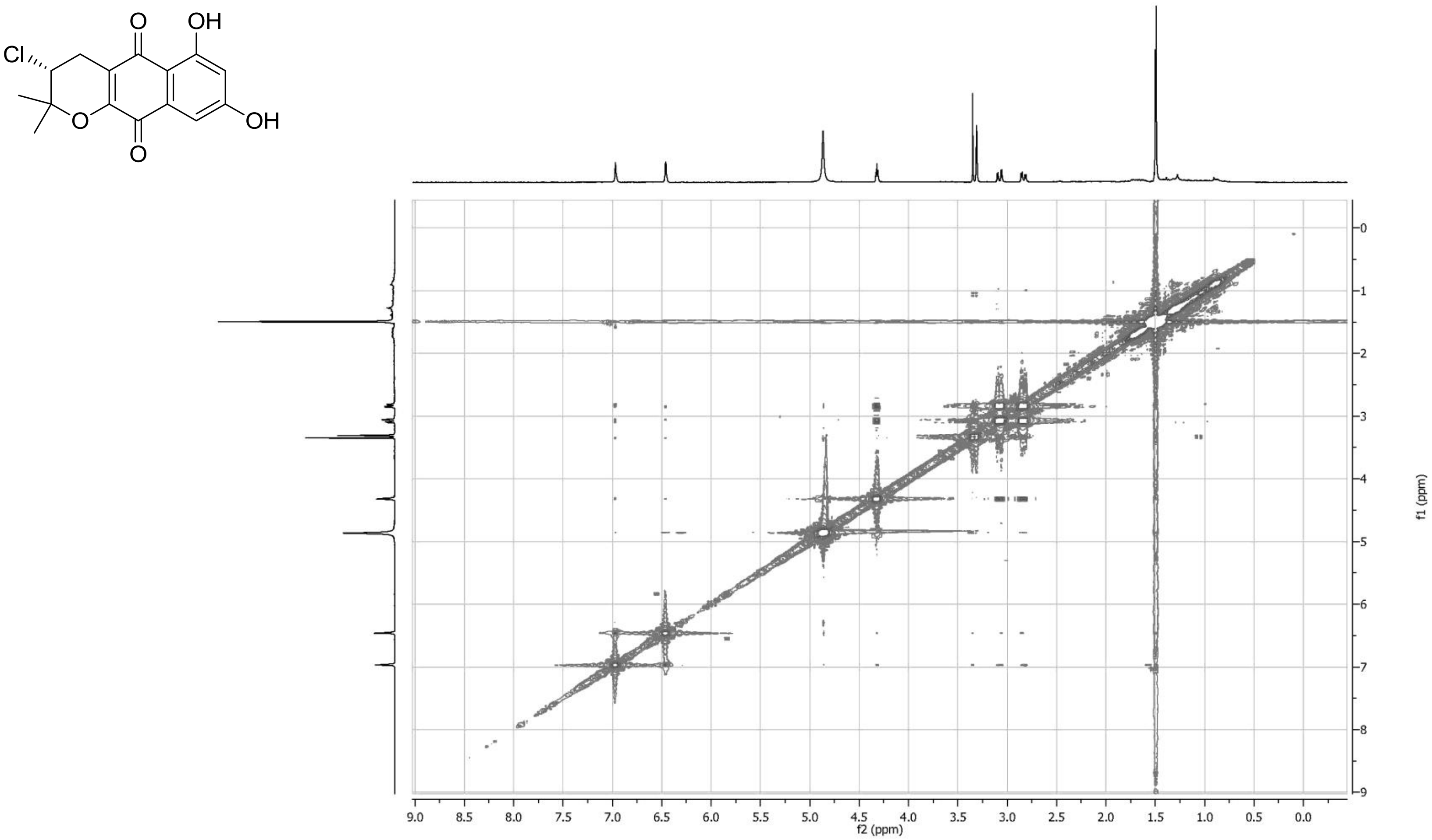

Figure S5. (**A**) The ^{1}H NMR spectrum of compound **3** measured in $CDCl_3$.

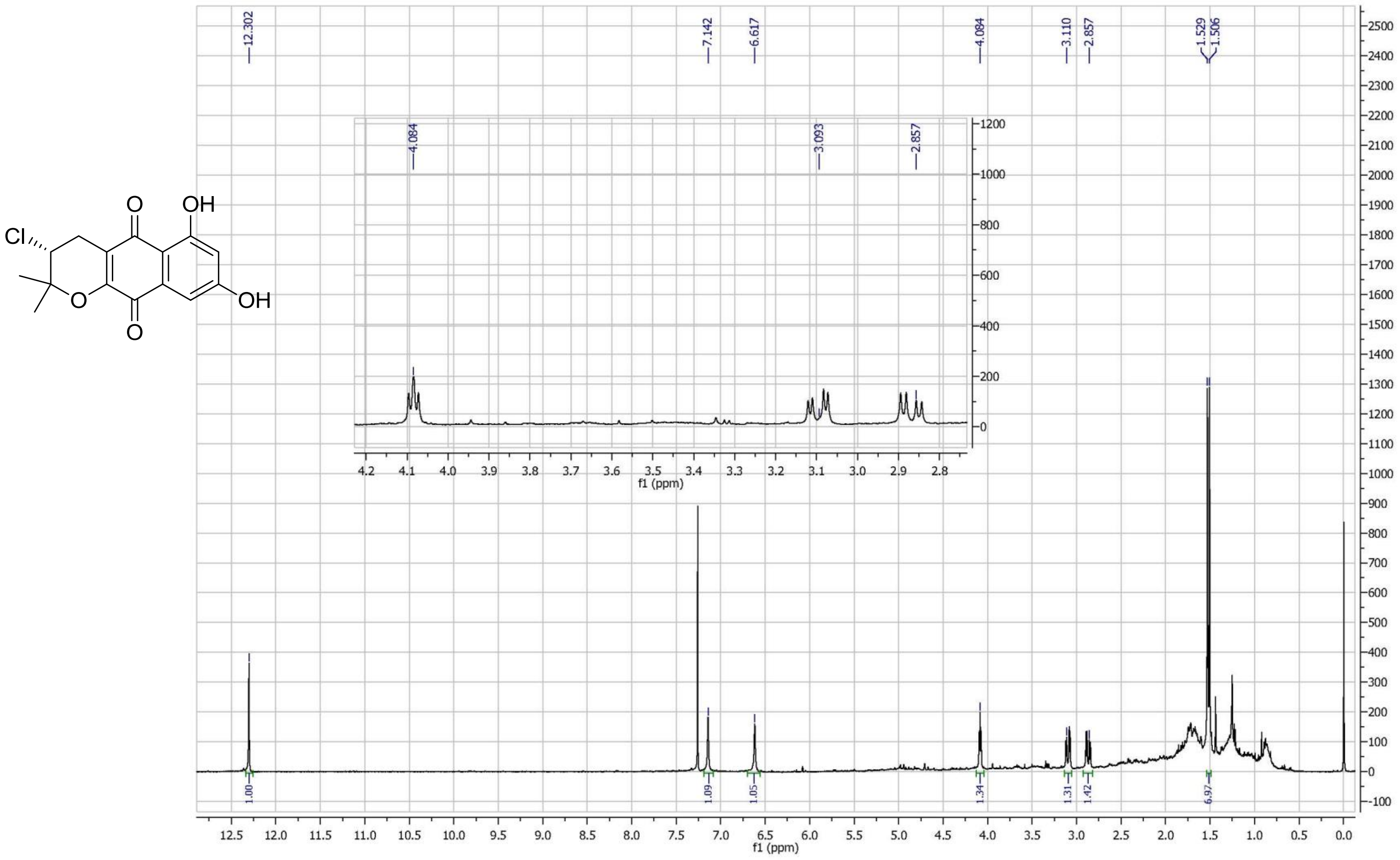

Figure S5. (**B**) The ^{13}C and the DEPT 135 NMR spectrum of compound **3** measured in $CDCl_3$.

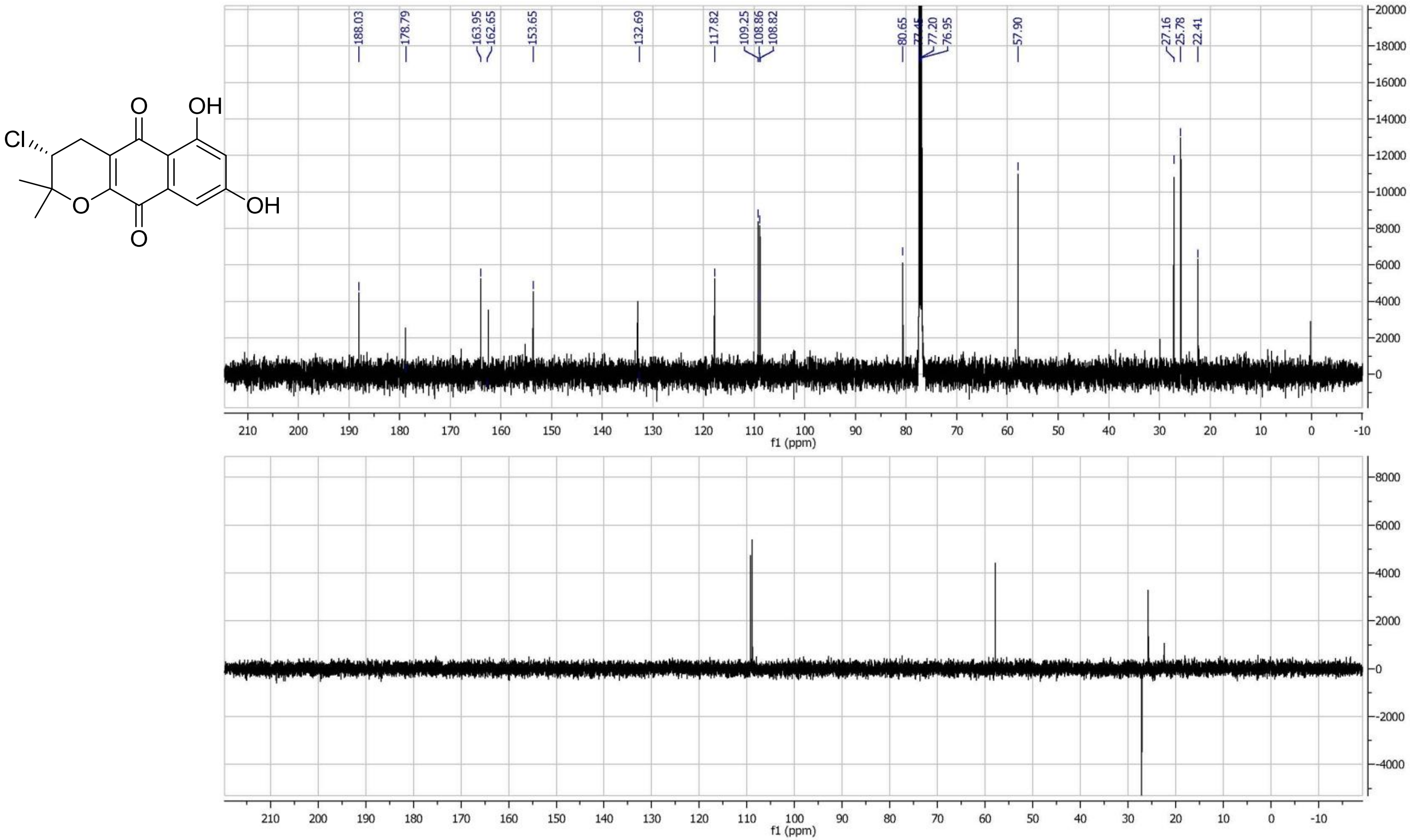

Figure S5. (**C**) The HMBC spectrum of compound **3** measured in $CDCl_3$.

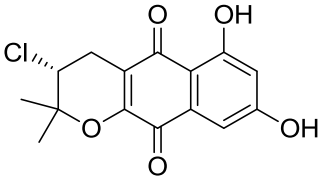

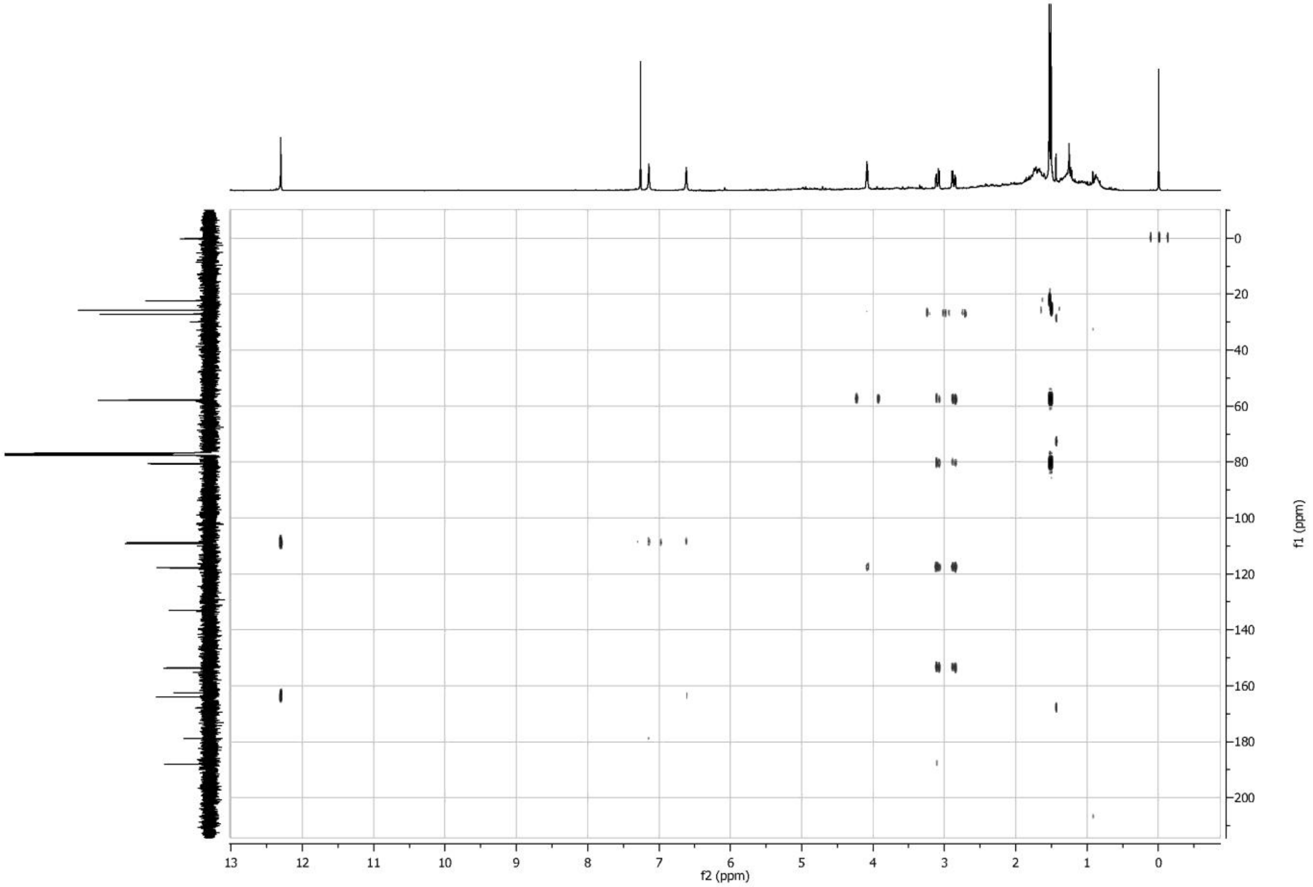

References

1. Shiomi, K.; Iinuma, H.; Hamada, M.; Naganawa, H.; Manabe, M.; Matsuki, C.; Takeuchi, T.; Umezawa, H. Novel antibiotics napyradiomycins. Production, isolation, physicochemical properties and biological activity. *J. Antibiot.* **1986**, *39*, 487–493.
2. Shiomi, K.; Nakamura, H.; Iinuma, H.; Naganawa, H.; Isshiki, K.; Takeuchi, T.; Umezawa, H. Structures of new antibiotics napyradiomycins. *J. Antibiot.* **1986**, *39*, 494–501.
3. Motohashi, K.; Sue, M.; Furihata, K.; Ito, S.; Seto, H. Terpenoids produced by actinomycetes: napyradiomycins from *Streptomyces antimycoticus* NT17. *J. Nat. Prod.* **2008**, *71*, 595–601.
4. Henkel, T.; Zeeck, A. Secondary metabolites by chemical screening. 15. Structure and absolute configuration of naphthomevalin, a new dihydro-naphthoquinone antibiotic from *Streptomyces* sp. *J. Antibiot.* **1991**, *44*, 665–669.

© 2013 by the authors; licensee MDPI, Basel, Switzerland. This article is an open access article distributed under the terms and conditions of the Creative Commons Attribution license (http://creativecommons.org/licenses/by/3.0/).
